# A Compendium of Mutational Signatures of Environmental Agents

**DOI:** 10.1016/j.cell.2019.03.001

**Published:** 2019-05-02

**Authors:** Jill E. Kucab, Xueqing Zou, Sandro Morganella, Madeleine Joel, A. Scott Nanda, Eszter Nagy, Celine Gomez, Andrea Degasperi, Rebecca Harris, Stephen P. Jackson, Volker M. Arlt, David H. Phillips, Serena Nik-Zainal

**Affiliations:** 1 Department of Analytical, Environmental and Forensic Sciences, MRC-PHE Centre for Environment and Health, King’s College London, 150 Stamford Street, London SE1 9NH, UK; 2 Academic Department of Medical Genetics, School of Clinical Medicine, University of Cambridge, Cambridge CB2 9NB, UK; 3 MRC Cancer Unit, University of Cambridge, Cambridge CB2 0XZ, UK; 4 Wellcome Trust Sanger Institute, Hinxton CB10 1SA, UK; 5 The Gurdon Institute, University of Cambridge, Cambridge CB2 1QN, UK

**Keywords:** Mutational signatures, mutagenesis, DNA damage, environmental mutagens, carcinogens

## Abstract

Whole-genome-sequencing (WGS) of human tumors has revealed distinct mutation patterns that hint at the causative origins of cancer. We examined mutational signatures in 324 WGS human-induced pluripotent stem cells exposed to 79 known or suspected environmental carcinogens. Forty-one yielded characteristic substitution mutational signatures. Some were similar to signatures found in human tumors. Additionally, six agents produced double-substitution signatures and eight produced indel signatures. Investigating mutation asymmetries across genome topography revealed fully functional mismatch and transcription-coupled repair pathways. DNA damage induced by environmental mutagens can be resolved by disparate repair and/or replicative pathways, resulting in an assortment of signature outcomes even for a single agent. This compendium of experimentally induced mutational signatures permits further exploration of roles of environmental agents in cancer etiology and underscores how human stem cell DNA is directly vulnerable to environmental agents.

**Video Abstract:**

## Highlights

• 41 of 79 environmental agents yielded substitution signatures
• 6 agents produced double-substitution signatures and 8 produced indel signatures
• Several signatures match or exhibit similarity with signatures found in human tumors
• Topographical mutational asymmetries reveal mechanistic insights

## Graphical Abstract

**Figure:**
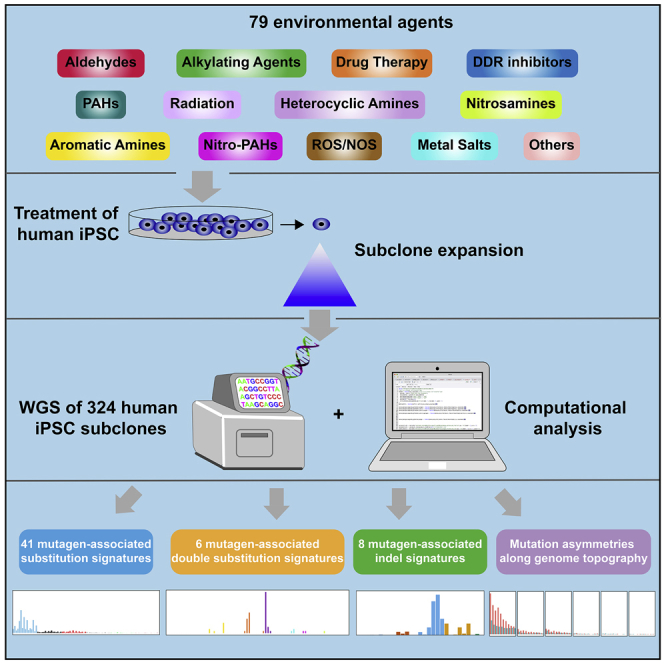

## Teaser

The effects of a range of environmental mutagens in terms of the kinds of mutations they induce and how these are repaired by the cell is presented in the form of a resource.

## Introduction

English physicians of the 18^th^ century are credited with linking environmental exposures to cancer. They observed an increased incidence of nasal polyps among users of snuff and associated scrotal cancer in chimney sweeps with chronic exposure to soot (Brown and Thornton, 1957). A century later, public health recommendations for frequent bathing for sweeps had seen the virtual eradication of scrotal cancer among sweeps in mainland Europe, but not in England, where bathing frequency remained low (Butlin, 1892). Subsequent associations between environmental agents and tumorigenesis include tobacco smoking and lung cancer, aniline dyes and bladder cancer, asbestos and mesothelioma, aflatoxin and liver cancer, and benzene and leukemia (Pfeifer et al., 2002, Walker and Gerber, 1981, Yang, 2011). The public health impact of understanding these associations is significant; identifying causes of cancer is essential for effective preventative interventions.

Although mechanisms underpinning how environmental carcinogens cause cancer are not fully understood, many cause DNA damage that results in mutations, as demonstrated experimentally in reporter genes (e.g., *lacZ*) and cancer-related genes (e.g., *RAS* and *TP53*) (DeMarini et al., 2001, Giglia-Mari and Sarasin, 2003, Pfeifer, 2000, Zhivagui et al., 2017). Specific patterns associated with exposure to particular carcinogens have been identified in *TP53* in human cancers too (Hollstein et al., 1991, Olivier et al., 2010), revealing that codon position, sequence context, and strand bias can be tumor-type- and carcinogen-specific. For instance, lung tumors from smokers harbor C > A/G > T transversion mutations in *TP53* codons 157, 158, 245, 248, and 273 (Pfeifer, 2000). Further, guanines at these codons were preferentially adducted and mutated in cells treated with benzo[*a*]pyrene-7,8-dihydrodiol-9,10-epoxide (BPDE), a reactive metabolite of the polycyclic aromatic hydrocarbon (PAH) benzo[*a*]pyrene (BaP) from tobacco-smoke in human (Denissenko et al., 1996) and mouse embryo fibroblast (MEF) models (Kucab et al., 2015). Additionally, G > T transversions induced *in vitro* and those in lung cancers exhibit a strong transcriptional strand bias. This is believed to reflect transcription-coupled nucleotide excision repair (TC-NER) of bulky adducts formed by tobacco carcinogens (Hainaut and Pfeifer, 2001).

Similar observations were made with other environmental exposures. UV light induces C > T/G > A and CC > TT/GG > AA transitions in DNA reflecting the formation of pyrimidine dimers (Pfeifer et al., 2005). This was corroborated by observations in UV-associated squamous and basal cell carcinomas and malignant melanomas. Aristolochic acid I (AAI), a phytochemical associated with urothelial cancer development (Nedelko et al., 2009), induces A > T/T > A transversions *in vitro*, as found in *TP53* in AAI-treated Hupki MEFs, mimicking the mutational spectra seen in urothelial tumors from patients exposed to aristolochic acid (Nedelko et al., 2009, Stiborová et al., 2016). These studies based on single gene analyses are highly informative but are limited by the fact that only a single mutation per sample was incorporated into each dataset.

Today, technological improvements permit whole genomes to be sequenced in a single experiment. Whole-genome sequencing (WGS) of a single malignant melanoma and a single lung cancer cell line first illustrated the power of this approach (Pleasance et al., 2010a, Pleasance et al., 2010b), revealing the characteristic mutational spectra of UV light and tobacco carcinogens, respectively. Subsequently, WGS of large numbers of other tumors revealed mutational patterns (Nik-Zainal et al., 2012a, Nik-Zainal et al., 2012b) in nearly all tumors (Alexandrov et al., 2013, Helleday et al., 2014) that arise from both endogenous and exogenous sources (Helleday et al., 2014, Nik-Zainal et al., 2016). Global, unbiased depiction provided by WGS has permitted more refined insights into mutational processes of human cancers, facilitating clinical applications of cancer genomics (Berger and Mardis, 2018, Mardis and Ladanyi, 2016).

Human cancers, however, result from environmental and endogenous exposures that are uncontrolled and in highly variable genetic backgrounds. Although mathematical methods have been applied to deconstruct mutation profiles into individual mutational signatures, these approaches are complex and fraught with issues of interpretation due to lack of experimental controls (Nik-Zainal and Morganella, 2017).

An important next step, therefore, is to systematically examine mutational patterns associated with a broad selection of environmental or therapeutic mutagens, generated under highly controlled conditions. We used a human induced pluripotent stem cell (iPSC) line, having the advantages of being normal, undifferentiated, fast-growing, and easy to clone. Most of the agents tested are classified by the International Agency for Research on Cancer as known, probable, or possible human carcinogens (group 1, 2A, and 2B, respectively). We present a first comprehensive assessment that we hope will serve the community in due course.

## Results

**Figure 1:**
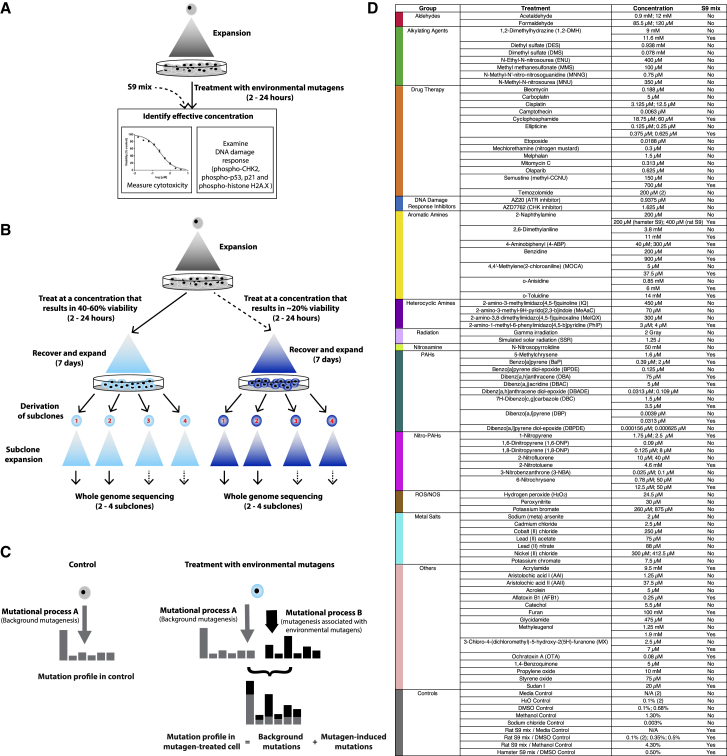
Experimental Protocol and Mutagen Information (A) Assessment of cytotoxicity and DNA damage response to identify effective concentrations. (B) Experimental workflow. (C) Schematic showing how a mutagen-associated mutational process changes a mutational profile. (D) List of mutagens and their treatment conditions. See also Figure S1 and Table S1.

This study included 77 chemical carcinogens, therapeutic agents, or DNA damage response (DDR) inhibitors, 2 sources of radiation, and a range of controls. These diverse agents damage DNA in various ways and may be repaired by different pathways. We assessed cytotoxicity and functional DDR readouts, subsequently generating a series of treated and control parental cell cultures (128 in total). From these, we derived single-cell daughter subclones (324 in total) and examined mutational patterns by WGS (Figure 1).

### Cytotoxicity and DNA Damage Response

To standardize treatment regimens, we used a concentration or dose for each agent that caused 40%–60% cytotoxicity, measured 72 h after treatment. For some treatments (n = 19), a higher concentration causing >80% cytotoxicity was also used (Table S1). Most chemicals had an IC_50_ in the μM range; 15 were in the mM range and 23 were sub-μM (Figure 1; Table S1).

Many compounds require metabolic activation into reactive intermediates to exert DNA damaging effects, often via cytochrome P450 enzymes. Because metabolic competence of iPSCs has not been established, 28/77 agents were tested with inclusion of S9 rodent liver-derived metabolic enzyme mixture.

**Figure S1:**
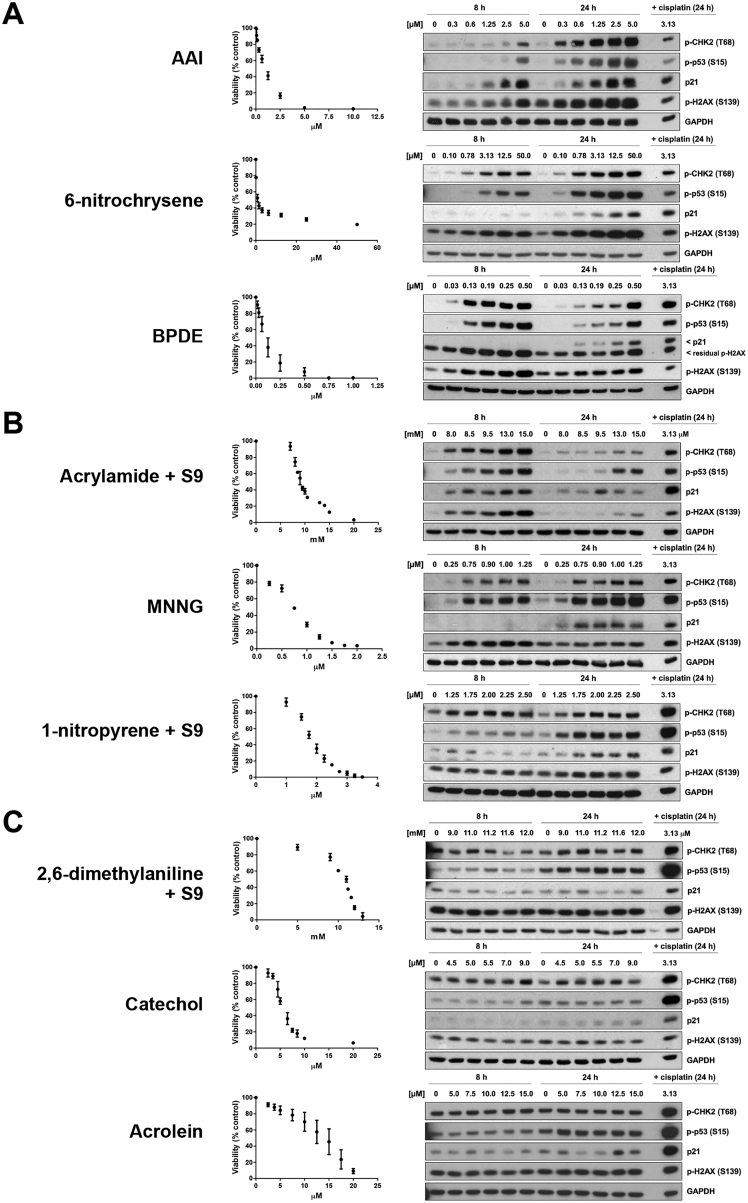
Cell Viability and DDR Induction following Treatment of Human iPSCs with Environmental Mutagens, Related to Figure 1, Table S1, and STAR Methods Included are examples of agents that (A) induced DDR and had an associated mutational signature, (B) induced DDR but did not have an associated mutational signature and (C) did not induce DDR and did not have an associated mutational signature. For viability assessment, cells were treated with the indicated concentrations of mutagen (or solvent control) for either 2 h (BPDE, DBADE and DBPDE), 3 h (when rat liver S9 mix was included) or 24 h. Viability was measured 72 h following initiation of treatment using the Deep Blue Cell Viability Kit. Mean values are shown as % of control ± SD of at least 3 replicate experiments. Protein expression of p-CHK2 (T68), p-p53 (S15), p21 and γ-H2AX (S139) was assessed by western blotting. GAPDH served as a loading control. Cells were treated as described above and lysed at 8 h or 24 h following initiation of treatment.

Induction of phosphorylation or expression of four DDR proteins was examined: phospho-CHK2, phospho-p53, p21, and γ-H2AX (Figure S1; Table S1). Sixteen of 113 treatment conditions (e.g., formaldehyde, catechol, acrolein) failed to induce detectable DDR signaling markers. Two of the 16 were associated with a mutation pattern (formaldehyde and 1,2-dimethylhydrazine [1,2-DMH] +S9). Of remaining treatments that induced one or more DDR markers, 51/97 were associated with mutation patterns. Intriguingly, acetaldehyde, N-methyl-N′-nitro-nitrosoguanidine (MNNG), and acrylamide induced DDR, but not detectable mutation patterns. Thus, the ability to induce DDR was not necessarily indicative of mutagenic potential.

### Identifying Mutational Signatures

A total of 324 subclones were derived from 128 control and mutagen-treated cultures; 62 had two subclones, 64 had three subclones and 2 had four subclones (Table S2). All were successfully sequenced to ∼30-fold depth. Short-read sequences were aligned to human reference genome assembly GRCh37/hg19. All classes of somatic mutations were called in subclones subtracting on the primary iPSC parental clone.

**Figure S2:**
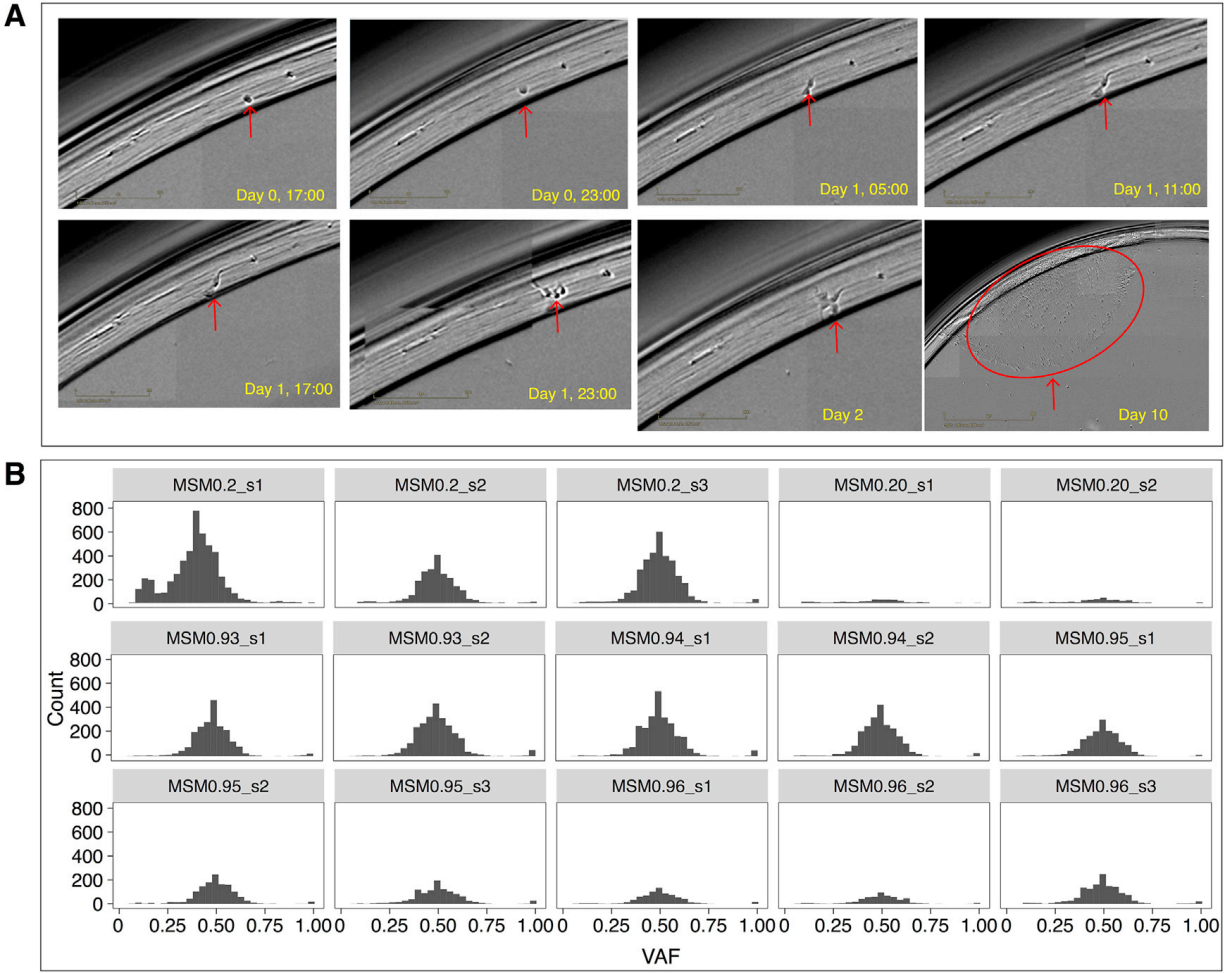
Using an IncuCyte to Follow Single-Cell-Derived Subclones, Related to STAR Methods (A) Screening images taken every 6 hours over 10-12 days. (B) Variant allele frequency of subclones, 15 randomly selected subclones are shown. To remove mutations present from potential polyclonal samples, a filter of VAF > = 0.2 was applied to substitutions and indels.

To ensure that the iPSC model remained stable and did not develop overt malignant potential, we evaluated chromosomal copy number in all 324 subclones. All remained diploid, unchanged from their parent. We looked for evidence of selection, including clonal and subclonal mutations in all DNA repair genes and in *TP53*, and for driver amplifications in all samples. None were identified. To ensure that we had comparable WGS data not arising from mixed populations, all experimental single-cell bottlenecks were monitored using an IncuCyte (Figure S2). Variant allele fraction distributions for all subclones were examined (STAR Methods) giving confidence that each mutational profile came from a single cell.

**Figure 2:**
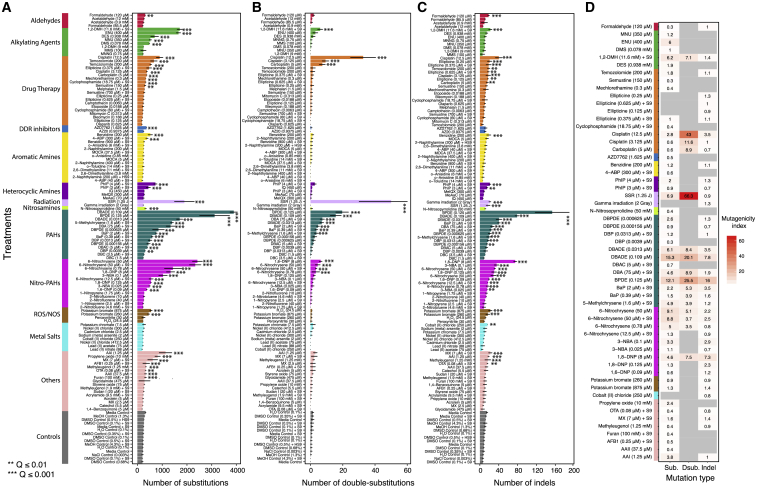
Mutation Frequencies (A–C) *De novo* mutation numbers identified for substitutions (A), double-substitutions (B), and indels (C). Asterisks indicate a significant increase in mutations over controls (STAR Methods). ^∗∗^q value ≤ 0.01; ^∗∗∗^q value ≤ 0.001 (permutation test). Data are for 2–4 independent subclone experiments. Bars represent mean ± SEM of subclone observations. (D) For treatments with a significant increase in mutations, mutagenicity index = (*N*_treatment_ − *N*_control_)/*N*_control_, where *N*_treatment_ is the average mutation number of treatment subclones and *N*_control_ is the average mutation number of control subclones. See also Tables S2 and S3.

Common culture reagents could generate mutational signatures and potentially confound interpretation of mutagen treatments. Thus, we included fifteen control clones treated with solvent concentrations matching those of the mutagen-treated samples; two were treated with water, three with culture media (one +S9 and two −S9), one with sodium chloride (0.003%), two with dimethylsulfoxide (DMSO) (at 0.68% and 0.1%), one with hamster S9 and 0.5% DMSO, four with rat S9 mix and DMSO at different concentrations (0.5%, 0.35%, and two at 0.1%), and two with methanol (4.3% with rat S9 and 1.3% without S9). All controls had a similar signature and level of background mutagenesis (∼245 substitutions, ∼1 double substitution, ∼10 short insertions/deletions [indels], and ∼0 rearrangements per genome) (Figure 2; Tables S2 and S3). Background mutagenesis has been reported in other human cellular systems (Behjati et al., 2014, Rouhani et al., 2016) and attributed to DNA damage incurred during cell culture.

**Figure 3:**
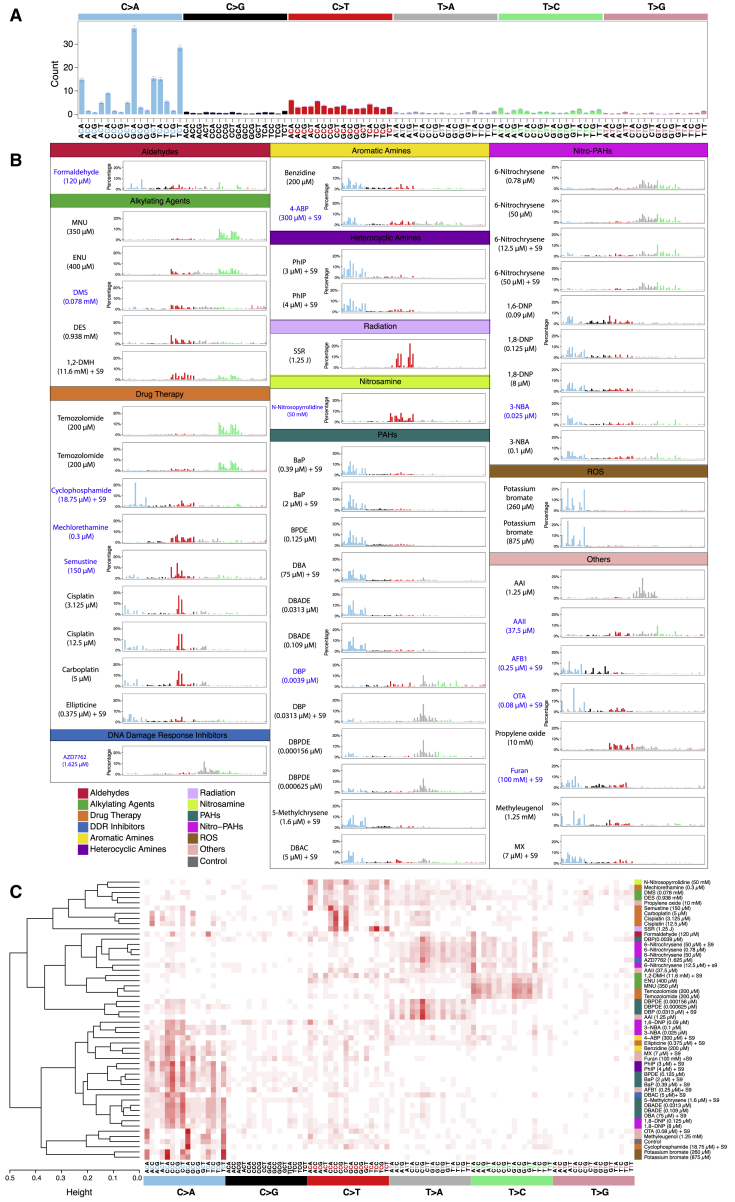
Substitution Signatures (A) Mutational profile of all controls. It is a 96-channel vector (6 types of substitution ^∗^ 4 types of 5′ base ^∗^ 4 types of 3′ base). Mean ± SEM of 35 subclones. This is also the background signature seen in all treatments. (B) Signatures identified from 53 treatments. Blue indicates a less stable signature (less consistent in subclones due to low numbers). (C) Hierarchical clustering of the 53 signatures. See also Figure S3 and Table S4.

Additional mutagenesis above background was variable between different treatments and consistent between subclones of the same treatment (Figures 2A–2C). To ensure systematic, robust signature discovery, we defined the ubiquitous background signature based on control samples and ensured that the total number of mutations for any given treatment was significantly greater than the controls (q value <0.01, permutation test). An index of mutagenicity was calculated to quantify effect size of mutagenesis over background (Figure 2D). We next determined if the mutational profile of a treatment was compellingly dissimilar to the background signature (signal-to-noise-ratio [SNR] > = 2; STAR Methods). Finally, we calculated a “stability” measure that penalizes excessive variation between subclones for a given treatment. This highly conservative additional step separates signatures of which we are strongly confident from those likely to be present but that we have less confidence in. For treatments that we considered had associated mutational signatures, the background culture-associated signature (Figure 3A) was subtracted, leaving a putative treatment signature (Figure 3B for substitutions; STAR Methods). To ensure that experimentally generated signatures were not due to a DNA repair defect acquired during culture, we searched for coding sequence mutations in subclones that could potentially influence mutational outcomes and found none of consequence.

**Figure 4:**
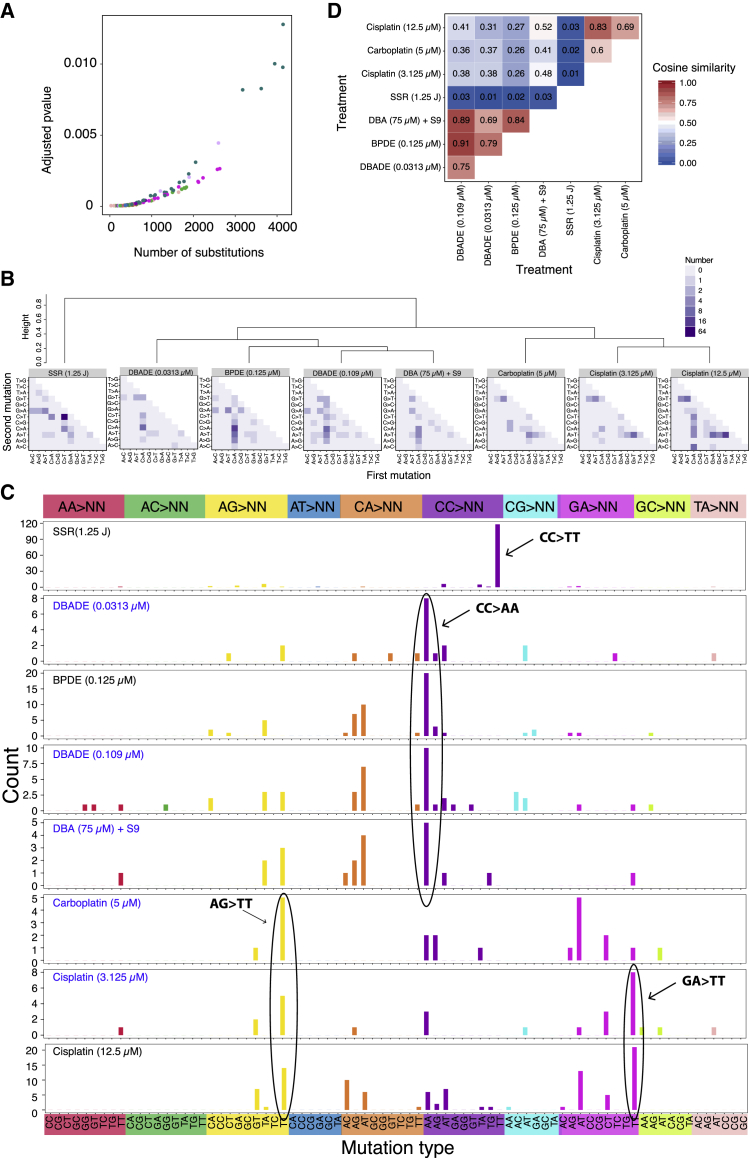
Double-Substitution Signatures (A) Expected probability of formation of a double-substitution by two random substitutions. (B) Hierarchical clustering of eight aggregated double-substitution profiles (treatments with double-substitution number > 20). The first mutation represents 5′ base change, the second mutation represents 3′ base change. In total, there are 78 types of double-substitutions (STAR Methods). (C) Double-substitution profiles as bar plots. Blue indicates a less stable signature. (D) Cosine similarities between eight double-substitution signatures. See also Table S6.

**Figure 5:**
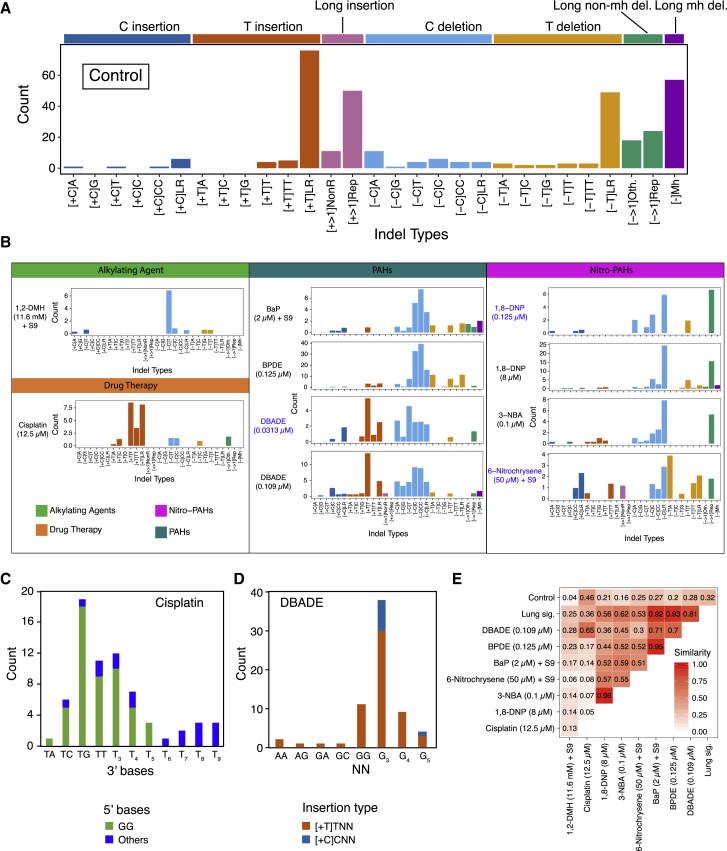
Indel Signatures (A) Indel profile of controls. Due to low numbers, all control subclones are aggregated to obtain a more accurate indel profile (see Figure S4). (B) Profiles of eight mutagens (10 treatments). Blue indicates less stable signature. (C) High resolution profile of cisplatin (12.5 μM)-induced one-base T insertion in repetitive sequence, taking 5′ sequence context into account. (D) High resolution profile of DBADE-induced T and C insertions. (E) Cosine similarities between ten mutagen, smoking-associated lung, and control signatures. See also Figure S4 and S5.

**Figure 6:**
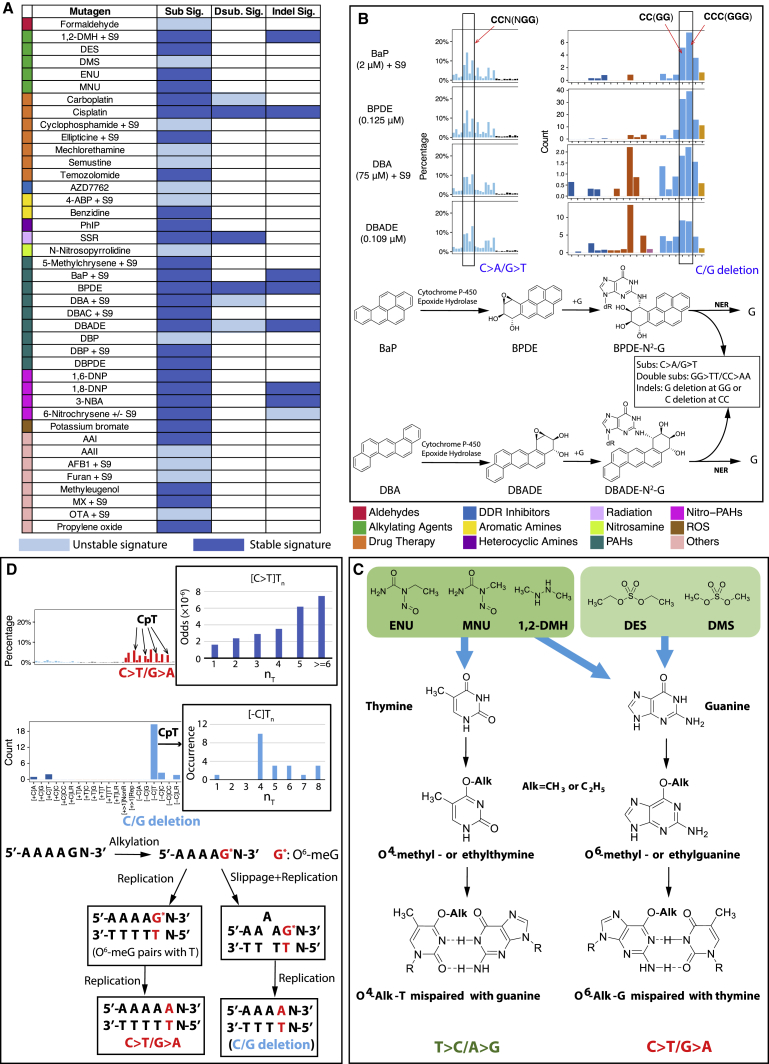
Mechanisms of Mutagen-Associated Mutational Signatures (A) Summary of mutagen-associated signatures. Light blue indicates an unstable signature (subclone variation); dark blue indicates a stable signature. (B) Sequence context of BaP, BPDE, DBA and DBADE substitution and indel mutation patterns. Substitutions and indels are more likely to occur near CC (GG). Pathways to BaP, BPDE, DBA, and DBADE mutations are shown. (C) Progression to mutation by five alkylating agents. (D) Proposed mechanisms underpinning 1,2-DMH substitution and indel signatures. 1,2-DMH alkylates Gs particularly at ApG sites. An increasing number of 5′A bases increases the probability of G mutating. Lower diagram: how signatures can arise for 1,2-DMH: in the left-hand branch, O^6^-meG in a (polyA)pG sequence pairs with T leading to a G > A substitution; in the right-hand branch, slippage additionally occurs resulting in a loss of T.

Of 113 treatment conditions involving 79 agents, approximately half induced additional numbers of substitutions and/or double-substitutions and/or indels, clearly different to controls (Figure 2). The numbers of rearrangements and copy number aberrations were limited and not informative. 53 putative single-base substitution mutational signatures were observed from 41 agents (Figure 3B), along with 8 double-substitution (Figure 4) and 10 indel signatures (Figure 5). Thus, distinct mutational signatures were seen for 52% of agents tested (Figures 2D and 6A; Table S2) with several producing more than one class of signature (Figure 6A).

### Substitution Mutational Signatures of Environmental Mutagens

**Figure S3:**
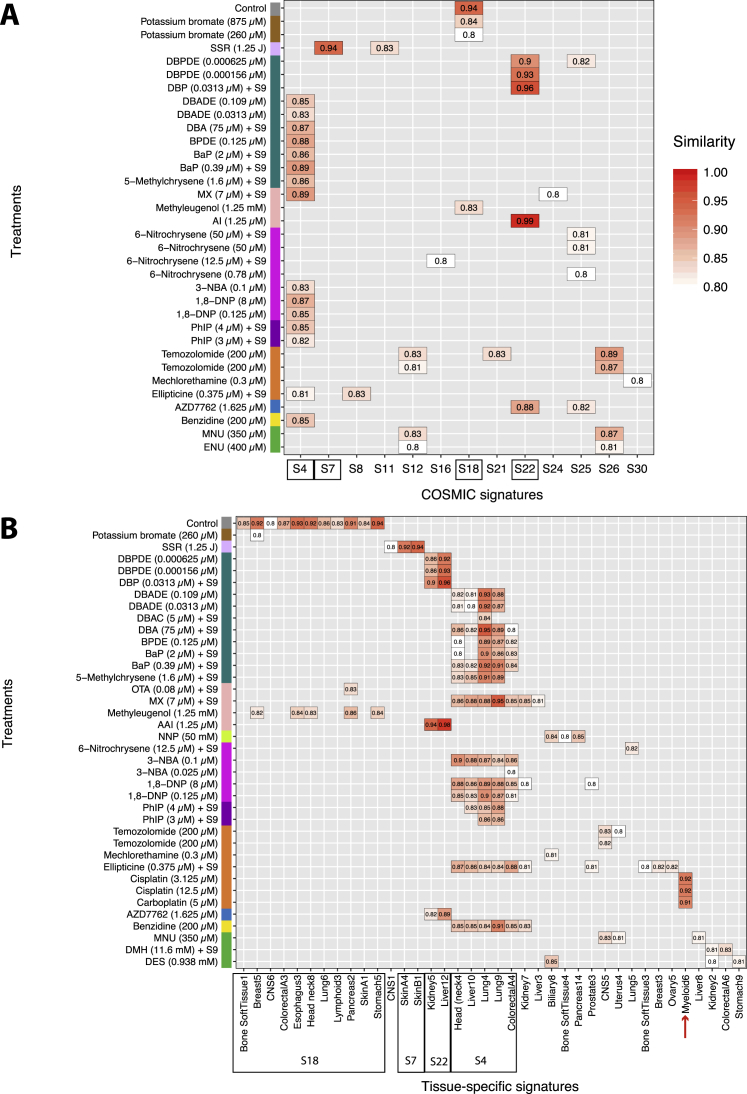
Comparison of Mutational Signatures between Cancer (*In Vivo*) and Mutagen (*In Vitro*), Related to Figure 3 (A) Cosine similarity between 30 COSMIC substitution signatures (https://cancer.sanger.ac.uk/cosmic/signatures) and mutagen substitution signatures. (B) Cosine similarity between tissue-specific substitution signatures and mutagen substitution signatures.

Our experiments detected some well-known mutational signatures. Simulated solar radiation (SSR) (Figure 3B) recapitulated the signature observed in UV-associated cancers (cosine similarity [henceforth cossim] 0.94) (Figure S3A) and UV-treated MEFs (Nik-Zainal et al., 2015) with 91% of SSR-induced substitutions being C > T/G > A transitions in our study. The mutational signature induced by AAI (Figure 3B) recapitulated that seen in urothelial cancers associated with aristolochic acid exposure (cossim 0.99) (Figure S3A) and in AAI-treated MEFs, dominated by A > T/T > A transversions.

28 mutagens were tested +S9, 13 of which were also tested −S9. Of the 15 tested +S9 only, 10 had signatures: 2-amino-1-methyl-6-phenylimidazo[4,5-b]pyridine (PhIP), cyclophosphamide, dibenz[*a,h*]anthracene (DBA), BaP, 5-methylchrysene, dibenz[a,j]acridine (DBAC), furan, 4-aminobiphenyl (4-ABP), aflatoxin B1 (AFB1), and ochratoxin A (OTA). Of 13 compounds tested, both +S9 and −S9, 6-nitrochrysene and dibenzo[*a,l*]pyrene (DBP) had signatures both ways, while 5 did not have signatures irrespective of state of metabolic activation (2-naphthylamine, 2,6-dimethylaniline, 4,4′-methylene(2-chloroaniline) [MOCA], 7H-dibenzo[c,g]carbazole [DBC] and o-anisidine). Three agents had signatures only when tested +S9 (1,2-DMH, ellipticine, 3-chloro-4-(dichloromethyl)-5-hydroxy-2(5H)-furanone [MX]). Benzidine, methyleugenol, and semustine had signatures only when tested −S9. In short, there was not a consistent impact of including an exogenous metabolizing system on mutagenesis; outcomes were compound-specific.

When a higher treatment concentration was also tested, 9 agents had near-identical signatures at both concentrations: 1,8-dinitropyrene (1,8-DNP), 3-nitrobenzanthrone (3-NBA), 6-nitrochrysene, BaP, dibenz[*a,h*]anthracene diol-epoxide (DBADE), dibenzo[*a,l*]pyrene diol-epoxide (DBPDE), PhIP, cisplatin, and potassium bromate. Three agents had slightly different outcomes at either concentration: cyclophosphamide, 4-ABP, and formaldehyde. For 6 compounds, higher concentration resulted in more mutations: DBADE, DBPDE, 1,8-DNP, 3-NBA, 6-nitrochrysene, and cisplatin. Six agents did not induce mutational signatures at either of two concentrations: 2-naphthylamine, 2-nitrofluorene, 2,6-dimethylaniline, acetaldehyde, nickel chloride, and DBC.

Next, we compared the 53 mutagen-induced substitution signatures by unsupervised hierarchical clustering of the 96-element profiles (Figure 3C). Reassuringly, several compounds with treatments performed at different concentrations clustered together, reflecting highly similar mutational profiles from independent experiments and the robustness of the system (e.g., 6-nitrochrysene, PhIP, 1,8-DNP, 3-NBA, and potassium bromate). Different compounds within the same family group bore likeness in mutational profile and clustered together (e.g., the alkylating agents temozolomide, N-methyl-N-nitrosourea (MNU), and N-ethyl-N-nitrosourea (ENU) and the platinum complexes cisplatin and carboplatin). Of interest, signatures of three PAHs, BaP, DBA, and DBP, bore closer similarity to those of their diol-epoxide metabolites (BPDE, DBADE, and DBPDE, respectively) than to each other.

There were also some surprises. DBP and DBPDE clustered closely with AAI. On closer inspection, there were striking similarities in the peaks of the T > A/A > T component between these different compounds reflecting a commonality in adduct formation at adenine residues by these disparate mutagens. In AAI, this transversion mutation accounted for 83% of the signature, whereas for DBP and DBPDE it amounted to 53%–70% of the total mutations. Thus importantly, different adducts can leave similar mutagenic imprints even when the primary mutagens are unrelated.

### Environmental Mutagens Cause DNA Damage Affecting Neighboring Nucleotides

Double-substitutions could arise due to two independent events occurring by chance at sites next to each other or when mutagenic damage at a site is linked to damage at the adjacent site. The latter is the case for CC > TT mutations caused by UV where modifications involving tandem pyrimidines result in 6,4-PPs and CPDs, as seen in *TP53* gene assays *in vitro* and in tandem *BRAF* mutations in malignant melanomas (Thomas et al., 2004).

The frequency of double-substitutions in our dataset (Figure 2B; Table S3) was higher than expected. To understand whether they arose because of elevated mutagenesis or because of the specific pattern of a given treatment, we performed simulation experiments correcting for mutation density and trinucleotide preponderance of experimentally induced mutational signatures and taking frequency of trinucleotides in the reference genome into account. The likelihood of observing even one double-substitution is small (Figure 4A). Thus, the model of two chance events causing double-substitutions is much less likely than one that postulates that there is an increased likelihood of affecting the mutability of the immediate neighboring base. Furthermore, we observed double-substitutions across all subclones of all treatments at a frequency higher than expected from the simulation experiments, including in control samples (Figure 2B). Thus, there may be a universal stressor increasing the likelihood of double-substitutions in the iPSCs.

Six agents (8 treatments) had statistically significant differences in double-substitution frequency compared with control (Figure 4B). Their patterns were diverse (Figures 4C and 4D). SSR was associated with a distinctive CC > TT pattern, in keeping with previous reports, constituting 6% of the total substitution burden in treated samples. Some of the PAHs (BPDE, DBA, and its metabolite, DBADE) tended to generate mainly CC > AA/GG > TT, but also CA > AT/TG > AT mutations (Figure 4C). Platinum compounds produced an AG > TT predominance, with GA > TT also observed (mainly restricted to cisplatin).

Double-substitutions were more likely to produce a TT outcome in all of the signatures identified (Figure 4C). This suggests that, regardless of primary DNA adduct, the most likely misreplication process that results in fixation of double-substitutions follows Strauss’ A-rule (Strauss, 2002), where A is inserted opposite an uninformative site, resulting in a subsequent T-fixed mutation. In conclusion, damage caused by particular environmental mutagens does have an effect on neighboring nucleotides. Whether these mutagens increase the likelihood of DNA adduct formation at adjacent nucleotides or increase the likelihood of erroneous repair of their neighbors is unclear.

### Environmental Mutagens Cause DNA Damage Resulting in Indel Signatures

Small indels (<100 bp) and substitutions arise through different mutational mechanisms. A model of strand slippage in repetitive DNA sequences creating misaligned intermediates with unpaired nucleotide loops was posited to be the preliminary step in indel formation (Fresco and Alberts, 1960, Streisinger et al., 1966, Streisinger and Owen, 1985). This is governed by post-replicative DNA mismatch repair (MMR) (Kunkel and Erie, 2005, Modrich and Lahue, 1996), reflected in excessively high 1–2 bp indel mutagenesis at polynucleotide repetitive sequences when MMR is inactivated (Greene and Jinks-Robertson, 1997, Tran et al., 1997) such as in colorectal carcinomas (Ionov et al., 1993, Thibodeau et al., 1993). By contrast, larger indels (≥3 bp in motif size) were noted to be enriched in cancers with mutations in *BRCA1*/*BRCA2* (Nik-Zainal et al., 2012a). These indels showed a small amount of homology between the indel motif and the flanking sequence, termed microhomology. The number of bases involved in microhomology was greater than expected by chance and believed to be the footprint of alternative non-homologous end joining (alt-NHEJ) double strand break (DSB) repair processes compensating for defective homologous recombination (HR) repair in tumors that are *BRCA1*/*BRCA2*-deficient. To call upon alt-NHEJ, the DNA damage step must have involved generation of one or more DSBs, thus representing an entirely different mutational process than that generating small indels at the polynucleotide tracts.

**Figure S4:**
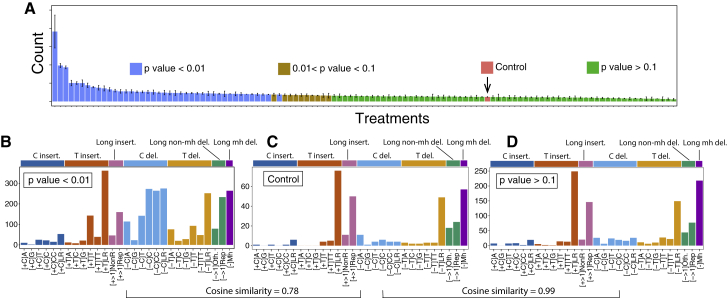
Identification of Background Indel Signatures, Related to Figure 5 and STAR Methods (A–D) Comparing indel number obtained from mutagen treatments and controls, one can identify the treatments that do not generate indel signatures (p value > 0.1). The aggregated control indel profile (bottom left) shows high similarity (0.99) with the aggregated indel profile from treatments with p value > 0.1 (treatments that do not manifest indel signatures, bottom right).

Analyses of indel mutagenesis have since taken flanking sequence characteristics into account. As many of the compounds used here have a preponderance to affect guanines, we extended indel classification to take nucleotide types into account. We categorized indels by class (deletion versus insertion), motif CG/TA content and size (1 bp or larger), CG/TA content of flanking sequence, and length of repetitive sequence if the motif was flanked by polynucleotide repeats. A microhomology-mediated category was restricted to deletions only. This resulted in 29 channels for indel signature discovery (Figure 5A and S4 for control indel profile; STAR Methods).

Indel signatures were obtained with 6 agents (8 treatments) (Figure 5B). 1,2-DMH had a unique preponderance for C deletions flanked by T nucleotides. In contrast, 1,8-DNP and 3-NBA, both nitro-PAHs, had near-identical indel signatures (Figure 5B), characterized by C deletions at long repetitive tracts and larger deletions (e.g., 2 bp and 3 bp motifs) at di- or tri-nucleotide repeat tracts. Another nitro-PAH, 6-nitrochrysene, had a more mixed indel phenotype. Cisplatin produced a mutational signature characterized by T insertions at single T or long tracts of repetitive Ts. These T insertions were just downstream of GpG dinucleotides (Figure 5C). This is in keeping with a previous report (Szikriszt et al., 2016) and is highly interesting because GpG dinucleotides are the targets of intrastrand crosslinks of platinum compounds.

**Figure S5:**
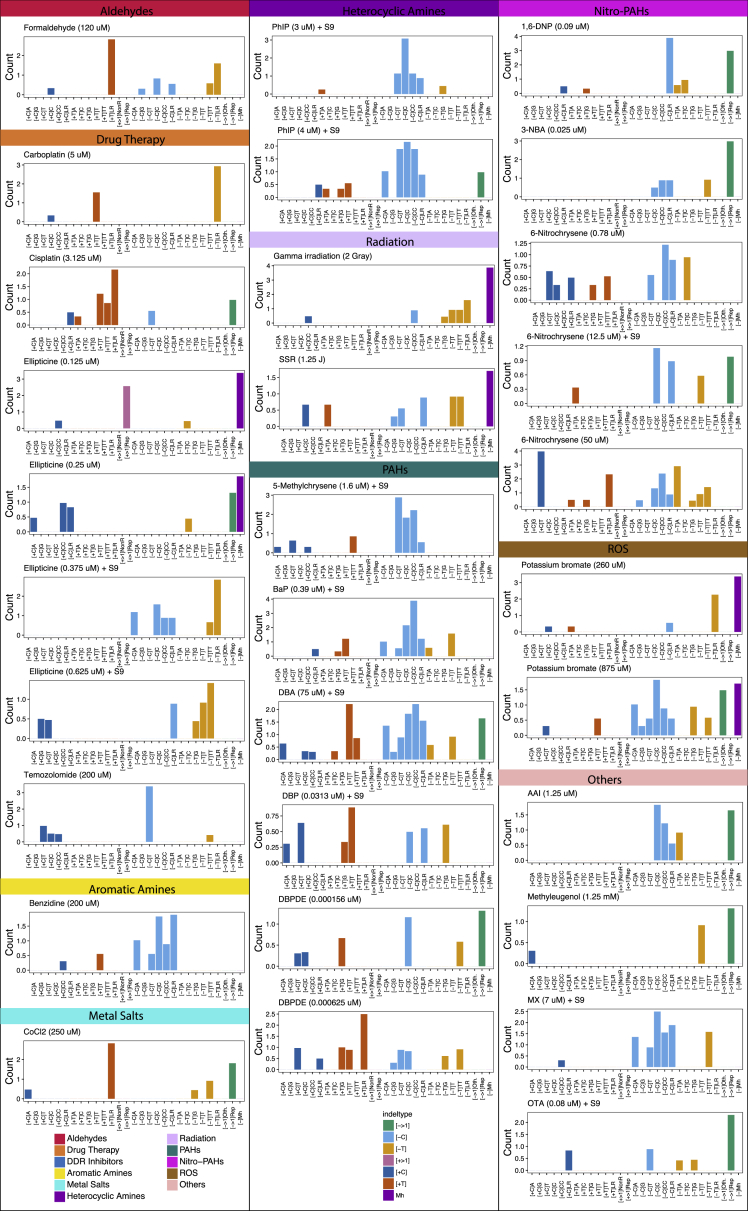
Indel Profiles of Mutagen-Associated Treatments with p Value < 0.01, Related to Figure 5 There are 41 treatments with a significant increase in indel numbers (P value < 0.01). Ten of them shown in Figure 5B have SNR > = 2, average indel number per subclone > = 20 and stability > = 0.7. The other 31 treatments did not show clear signatures, because the increased number of indels in each subclone was relatively low e.g., less than 10 above the baseline, and the number of subclones of each treatment is low (2-4). By distributing less than 20-40 indels into 29 channels, one is hardly able to appreciate a signature. Although we do not have enough power to obtain full pictures of indel signatures for these 31 treatments, some characteristic features are appreciable. For example, treatment of 3.125 μM cisplatin has T insertion in poly T tracks, which is similar to treatment of 12.5 μM cisplatin. Treatments of PhIP with S9 at two concentrations all show distinct C deletions. Two radiation experiments, namely gamma irradiation and simulated solar radiation, both show increased microhomology-mediated deletions, indicating additional double-stranded DNA breaks may be induced by radiations. For PAHs, treatment with 5-methylchrysene (1.6 μM) +S9 induced additional C deletions; treatment with BaP (0.39 μM) +S9 has a similar profile to treatment with BaP (2 μM) +S9 and BPDE (0.125 μM) (Figure 5B); treatment with DBA (75 μM) +S9 shows both increased T insertion and C deletion, similar to treatments with DBADE (0.0313 μM and 0.109 μM). It seems that many mutagens from different groups are able to cause C deletions, such as potassium bromate (875 μM), AAI (1.25 μM), MX (7 μM) with S9, 1,6-DNP (0.09 μM), 3-NBA (0.025 μM) and 6-nitrochrysene, indicating the damage on guanine can often result in C:G pair deletion. Thus for the cohort described in this paragraph, indel signatures may well exist, but according to our conservative criteria we did not report these as signatures because the current study is underpowered to be able to do this.

Among the PAHs, BaP, BPDE, and DBADE had strong resemblances to the indel signature extracted from smokers’ lung cancers (Figure 5E). BaP had an indel signature defined by deletions of C specifically at C repetitive sequences of <3 bp (i.e., C flanked by C or CC), nearly identical to its diol-epoxide, BPDE (cossim 0.95, Figure 5E). DBA also had an excess of C deletions at repeat sequences <3 bp but did not get called as a high-confidence signature (Figure S5). Its diol-epoxide DBADE showed an additional component of T insertions at single T nucleotides. Inspection of surrounding sequence downstream of the single 3′ T revealed a propensity to occur at G runs (Figure 5D). It is interesting to consider that repair of an adducted guanine could result in an indel in its vicinity. Last, DBADE had a stronger mutagenicity index for forming indels than substitutions over background, similar to its parent compound DBA (Figure 2D).

Thus, DNA damage induced by particular agents can be associated with multiple mutational signatures of different classes (Figures 2D and 6A). The diversity of mutational outcomes observed for some mutagens is likely to be due to different mechanisms of resolving a particular type of damage (e.g., a cisplatin intrastrand crosslink), which may involve different translesion polymerases.

### Mutational Signatures Derived from PAHs

PAHs are considered to be among the most significant mutagenic components of tobacco smoke. Of the fourteen treatments involving PAHs and related metabolites, all were associated with signatures apart from DBC (Figures 3B and 6A; Table S4).

BaP, DBA, and DBP clustered closer to their respective diol-epoxides than to one another in the hierarchical-clustering exercise. Although several mechanisms have been proposed for how PAHs exert their biological effects (IARC Working Group on the Evaluation of Carcinogenic Risks to Humans, 2010), the close similarity between the signatures of parent PAHs and their respective diol-epoxides is powerful evidence that the diol-epoxide pathway of metabolic activation is responsible for the mutagenic activity of the PAH class in mammalian cells. For BaP and BPDE, a high proportion of the mutations are at G:C pairs, and most are G > T/C > A, reflecting how their DNA adducts are formed principally at N^2^-guanine. Concordant with previous reports, the propensity for guanine damage is particularly elevated at methylated CpGs (p value < 0.01, Fisher exact test). In contrast, DBP and DBPDE signatures contain a majority of mutations at A:T, consistent with predominant DNA adduct formation at the N^6^-adenine.

5-Methylchrysene is activated via a diol-epoxide that reacts with guanine. 90% of its mutations are at G:C, mainly G > T/C > A (71%), with greatest similarity to DBA/DBADE (cossim 0.95 and 0.97, respectively). The distinguishing feature of the G > T/C > A transversions induced by these compounds is that the tallest peak is at CpCpT, followed by CpCpC. By contrast, for BaP and BPDE, the tallest G > T/C > A peak occurs at CpCpC followed by CpCpT. DBAC, which has a nitrogen atom in one of its 6-membered rings, clustered separately from the other PAHs and is most similar to 3-NBA, a nitro-PAH. Its signature comprises mainly G:C mutations (70%), of which 52% are G > T/C > A transversions.

Four compounds had significantly elevated numbers of substitutions, double-substitutions, and indels: BaP, BPDE, DBA, and DBADE. Each of these classes of signatures occurred at tracts of CC or CCC (equivalent to GG or GGG) (Figure 6B and S5). Thus, the primary DNA damaging step is adduct formation at short runs of guanines, but resolution of that damage, by the replicative A-rule, MMR and/or TC-NER, underpins the different final imprints (Figure 6B).

### Mutational Signatures Associated with Free Radicals

Free radical species induce a number of DNA lesions, the most abundant being 8-oxo-G, which can create G > T/C > A transversions, particularly at runs of consecutive guanines. Although hydrogen peroxide, anticipated to create ROS, and peroxynitrite, which generates reactive nitrogen (nitric oxide) species, did not yield clear mutation patterns, potassium bromate that generates hydroxyl radicals did show enrichment for G > T/C > A mutations (88%) in two independent experiments (260 μM and 875 μM). Its signature clusters separately but bears closest similarity to the cluster that includes the background signature that is similar to COSMIC Signature 18 (Figure S3A), reported to be due to ROS. Ochratoxin A and methyleugenol also clustered in this group, and their signatures may reflect an enhanced production of ROS (Figures 3C and S3A).

### Mutational Signatures of Alkylating Agents

Alkylating agents add an alkyl group (C_n_H_2n+1_) to DNA. O^6^-alkylguanine causes G > A/C > T, O^4^-alkylthymine causes T > C/A > G and O^2^-alkylthymine causes T > A/A > T, while N-alkylpurines (N7-dG and N3-dA) can give rise to apurinic sites (Jenkins et al., 2005) (Figure 6C).

Eight alkylating agents were examined (9 treatments overall—1,2-DMH was assessed +S9 and −S9). 1,2-DMH (−S9), methylmethanesulfonate (MMS), and MNNG were not associated with significant mutational patterns. MMS subclones had total numbers of mutations slightly above background but a nondescript mutational profile, not distinctive enough to be detected as a signature. MNNG has been reported to produce a characteristic C > T/G > A mutation pattern in MEFs (Olivier et al., 2014), but we did not see a signature in either of its subclones.

Five alkylating agents produced mutation patterns clustered in two distinct groups (Figure 3C). ENU and MNU clustered with temozolomide (listed as a drug therapy but is also an alkylating agent) with signatures dominated by T > C/A > G transitions (85%, 50%, and 78% respectively) and highly correlated with one another (cossims: temozolomide versus MNU 0.98, temozolomide versus ENU 0.84; MNU versus ENU 0.85). The other group comprising diethyl sulfate (DES) and dimethyl sulfate (DMS) additionally had C > T/G > A transitions (40% and 29%, respectively) and were similar to each other (cossim 0.80). 1,2-DMH produced roughly equal amounts of T > C/A > G (33%) and C > T/G > A (42%) and its signature did not resemble patterns produced by other alkylating agents. Of note, the temozolomide mutational pattern was not like COSMIC Signature 11 that had been previously associated with this treatment in neuro-oncology tumors. Instead, the C > T/G > A component of the mutation pattern of 1,2-DMH bears a striking resemblance to the C > T/G > A component of Signature 11 (cossim 0.89), tallest peaks at NpCpC and NpCpT, and is the only compound in this family to do so. The C > T/G > A component of DES and DMS is different with the tallest peaks at ApCpA, ApCpT, and CpCpT.

The predominance of T > C/A > G and C > T/G > A in the signatures of alkylating agents may be a consequence of DNA repair processes in iPSCs. O-alkylating DNA damage is usually repaired by direct reversal. Mammalian alkylguanine-DNA-transferases (AGTs) transfer the alkyl group from O-alkylated bases to a receptor cysteine residue with the AGT protein. The process is more efficient for O^6^-alkylguanine than for O^4^-alkylthymine and O^2^-alkylthymine (Jenkins et al., 2005). Thus, the signatures observed with ENU and MNU, which are more likely to form O-alkylations, may reflect a greater persistence of O-alkylthymine adducts that subsequently result in T > C/A > G transitions. By contrast, DMS and DES are stronger N-alkylators, and although they are also O-alkylators, O-alkylthymine has not been reported for this group (Jenkins et al., 2005). Hence, their signatures are possibly the consequence of mis-replication of persistent O^6^-alkylguanine mis-read as adenine with insertion of thymine on the opposite strand resulting in excess of C > T/G > A transitions.

Interestingly, 1,2-DMH presents an indel phenotype of C deletions often flanked by thymine. Of 21 such indels, 20 were at poly-T tracts of 4–8 bp (Figure 6D). Its substitution signature has an excess of C > T mutations at CpTs that are also enriched at poly-T tracts (Figure 6D). We posit that O^6^-alkylation by 1,2-DMH occurs on guanines at the end of poly-A tracts (equivalent to cytosines abutting poly-Ts). Damage resolution results in two different outcomes: O^6^-meG pairs with thymine resulting in a G > A/C > T substitution or its immediate neighbors (or indeed itself) are excised similar to an insertion-deletion loop resulting in a single nucleotide deletion (Figure 6D).

### Insights into Mutational Mechanisms of Platinum Complexes

The substitution signatures of the two platinum complexes were dominated by G > A/C > T with a high degree of similarity (cossim 0.95). The cisplatin signature was very similar to that in MCF10A and HepG2 cells (Boot et al., 2018). The G > A transitions occurred predominantly at GpGpG or ApGpG sequences (equivalent to C > T at CpCpC and CpCpT).

We also report double-substitution signatures of the platinum complexes, namely AG > TT and GA > TT. Cisplatin and carboplatin form intrastrand crosslinks at purines (e.g., ApG, GpA, GpG) and a mispairing of such crosslinks with AA (Strauss, 2002) would result in TT mutations. Thus, misreplication across uninformative sites may be the driving force behind fixation of double-substitutions.

Both compounds produced indel patterns, although only the cisplatin signature reached significance (Figure 5C). We postulate that error-prone excision repair of intrastrand crosslinks can result in “collateral damage” with indels of nearby nucleotides.

Accordingly, primary DNA damage associated with platinum complexes may be enhanced at GpG nucleotides but subsequent cellular attempts at resolving intrastrand crosslinks creates alternative mutagenic outcomes. Whether through crosslink repair, error-prone excision repair, or translesion synthesis, there are diverse signatures associated with platinum compounds.

### Mutagenesis by Other Compounds

Human exposure to plant extracts containing aristolochic acid involves exposure to at least two structurally related genotoxic compounds, AAI and aristolochic acid II (AAII). Both compounds gave rise to mutational signatures but only the former resembled that seen in human cancers associated with aristolochic acid exposure (Signature 22; cossim 0.99). This signature is highly reproducible across multiple human tumor sites (urothelial, liver) and at least two *in vitro* cellular systems (Nik-Zainal et al., 2015, Poon et al., 2015).

Four agents (cyclophosphamide, furan, N-nitrosopyrrolidine, and MX) form cyclic adducts with DNA bases, including 1,N^2^-dG, 3,N^4^-dC, and 1,N^6^-dA. However, their signatures were distinctly different. This may reflect differentially dominating adducts in each case or be because structurally dissimilar cyclic adducts formed at the same binary positions in DNA have different mutational consequences.

Overall our study has examples of agents with similar structures and/or mechanisms of action having similar signatures (cisplatin and carboplatin; temozolomide, MNU, and ENU; DMS and DES; DBA, BaP, and 5-methylchrysene), but also examples of distinctly different signatures arising from closely related compounds (1,6-DNP and 1,8-DNP; AAI and AAII). There are also intriguing examples of dissimilar agents with similar signatures: PhIP and BaP/BPDE (cossim 0.95); MX and benzidine (cossim 0.96); AAI and the TA-AT component of the DBP and DBPDE signatures (cossim 0.96).

### Relationships between Mutagen-Derived and Cancer-Derived Signatures

We compared our experimentally generated mutational signatures with those derived from human cancers. To ensure that the analysis was not biased by prior assumptions of pre-defined consensus mutational signatures, we revisited mutational signature extraction of 2,577 whole cancer genomes (unpublished data). Tissue-specific signature extractions were performed, identifying 196 independent signatures in 21 tissue types. We expect some signatures to be similar between cancer types (e.g., Signature 1 is age-associated and Signatures 2 and 13 are associated with APOBEC activity).

The strongest similarities observed are between the *in vitro* AAI signature and liver and kidney cancer signatures (cossim 0.98 and 0.94, respectively) (Figure S3B) associated with exposure to AAI. There was also concordance between DBP and DBPDE with the same signatures (range, 0.86–0.96) (Figure S3B). As mentioned earlier, this is likely because of similar presumptive adduct formation on adenines by these agents and not because of human exposure to DBP/DBPDE.

The SSR signature shows greatest resemblance to the UV signatures in skin tumors. Signatures from PAHs (DBA, DBADE, DBAC, BaP, BPDE, and 5-methylchrysene) show greatest similarity to the lung cancer signature associated with tobacco smoking (range, 0.84–0.95) (Figure S3B). Many chemical compounds likely contribute to the carcinogenicity of tobacco smoke. The contributions of each PAH to parts of COSMIC Signature 4 can be seen, including a distinct T > A/A > T peak at CpTpG, identical to that observed with DBP and DBPDE. We can now also attribute double-substitution and indel components of tobacco-smoke mutagenesis to BaP, DBA, and their diol-epoxides, BPDE and DBADE (Figures 4C and 5B).

Other signatures have weaker relationships with some cancer signatures and must be interpreted with caution. They include nitro-PAHs, alkylating agents, heterocyclic and aromatic amines, and drug therapies. Of interest, our cisplatin and carboplatin signatures show concordance with a signature extracted from myeloid tumors in patients that had received chemotherapy.

Last, the ubiquitous background signature present across the control samples is similar to COSMIC Signature 18, previously hypothesized to be due to ROS. Indeed, many cancer types also show Signature-18-like patterns. A signature associated with defective *MUTYH*, a glycosylase that excises 8-oxoG from DNA, has also been shown to strongly resemble Signature 18 (Pilati et al., 2017).

### Impact of DNA Repair on Mutational Signatures of Environmental Mutagens

The experiments were performed in a single cell line, so there was identical availability of DNA repair and/or replicative pathways for all treatments. We evaluated their mechanistic contribution to the mutational signatures through analyses of genome topography.

Despite considerable nucleotide level variation, there were high levels of chromosomal stability with few structural variants across all 324 daughter subclones. Therefore, in our system, DDR is robust with little tolerance for mutagenesis initiated by double-strand-breaks (DSB). This reinforces work by others exploring DNA repair pathways in stem cells that found DDR and DSB repair to be intact.

Replicative strand asymmetry was not observed in any signature. Replication is less likely to play an influential role in repair of exogenous damage, and more likely to impact intrinsic mutational processes, as evidenced by strong replication strand asymmetry seen for endogenous processes such as APOBEC-related mutagenesis and MMR deficiency (MMRd) (Morganella et al., 2016, Zou et al., 2018).

**Figure S6:**
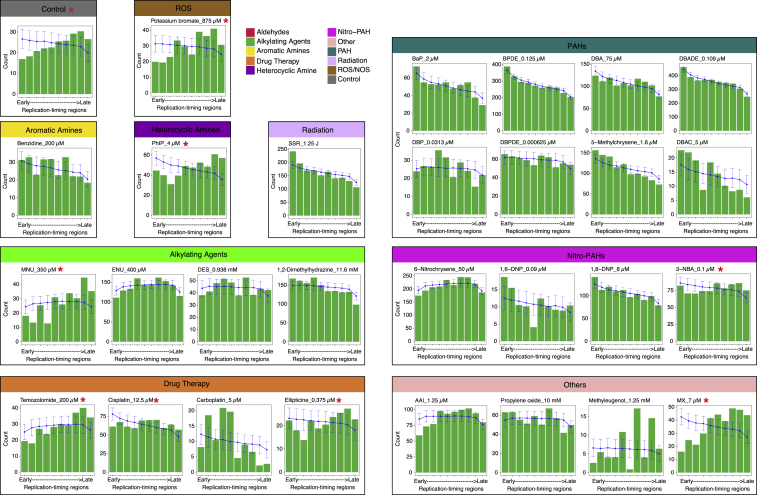
Distribution of Normalized Mutation Density across the Replication Timing Domains, Related to Figure 7 The G2/S phase was separated into ten replication timing domains. The expected distribution of mutations in replication timing regions was obtained through simulation based on the signature profile and trinucleotide distribution. Red asterisk “^∗^” marks the mutagen treatments having observed distribution (green bars) different from simulated distribution (blue line).

Across replication timing domains (RTD), mutation densities of many signatures mirrored their expected distribution when corrected for the reference genome trinucleotide content and for the sequence context predilection (Figure S6).

**Figure 7:**
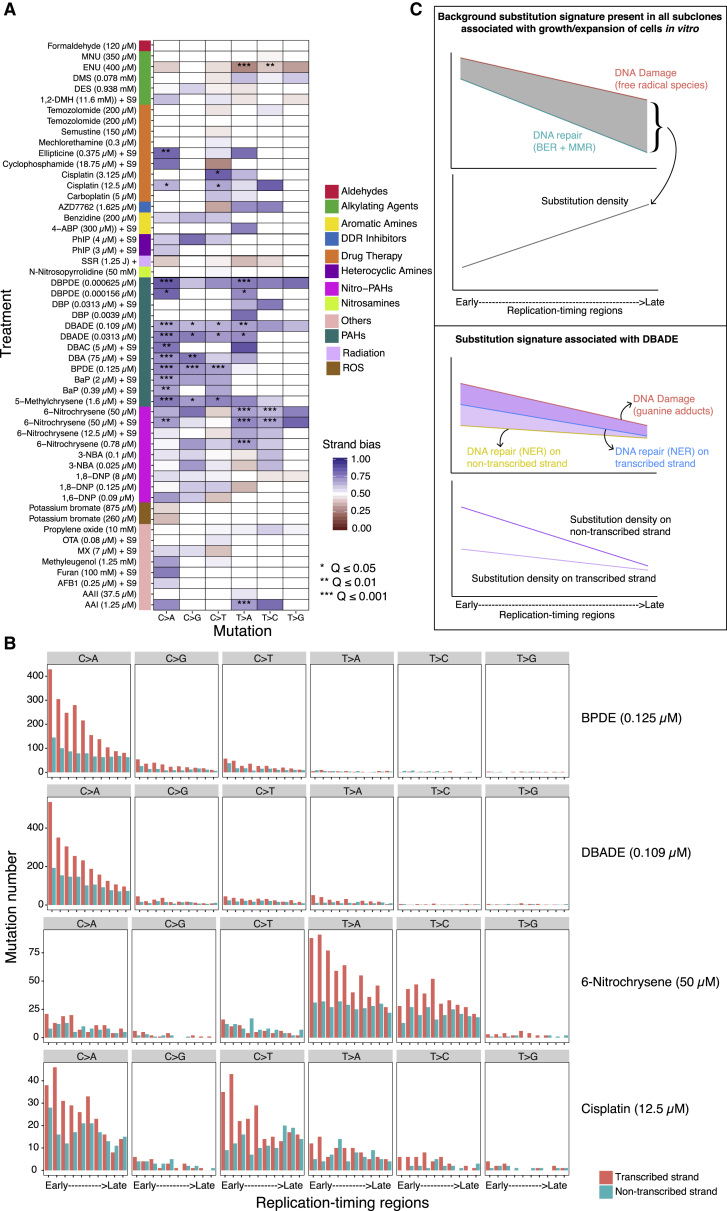
Strand Asymmetries and Genomic Distributions of Mutagen Signatures (A) Transcriptional strand asymmetry of 53 mutagen substitution signatures in 6 channels. Asterisks indicate significant bias. ^∗^q value ≤ 0.05; ^∗∗^q value ≤ 0.01; ^∗∗∗^q value ≤ 0.001. Pearson’s chi-square test with multiple test correction. (B) Transcriptional strand asymmetry across RTDs of four selected agents. (C) Schematic illustration on efficiency of DNA repair along RTD, contrasting mutagenesis during culture/expansion of cells *in vitro* mainly due to ROS (top) with mutagenesis caused by DBADE forming N^2^-G adducts (bottom). Guanine-associated DNA damage (red lines) is more likely to occur at GC-rich regions that tend to be enriched in early RTD. Hence, there is a negative gradient of an excess of damage in early RTDs for both of these forms of DNA damage, although high level of DBADE damage results in a steeper gradient. Fortunately, DNA repair is also often more efficient in early RTDs. BER and MMR contribute to the repair of guanine-associated damage caused by ROS (cyan line, top). Likewise, TC-NER is involved in the repair of DBADE-associated guanine adducts (blue and yellow lines, bottom). For the culture-related signature (top), BER and MMR must be operational and highly efficient particularly in the early RTD in order to achieve the distribution observed given by the difference between the red and cyan lines (gray zone). This results in a final distribution that has an excess of mutagenesis in late RTD in all subclones. For the DBADE signature, TC-NER must be fully operational because the gradient of substitutions is different between the transcribed (blue line) and non-transcribed strand (orange line), culminating in the mutational distributions across RTD shown in deep purple and light purple lines, respectively. The difference in substitution density between non-transcribed strand (deep purple line) and transcribed strand (light purple) is greater in early replication regions than in late ones. This is observed consistently for many PAHs and is also in cancer-derived signatures. See also Figures S6 and S7.

**Figure S7:**
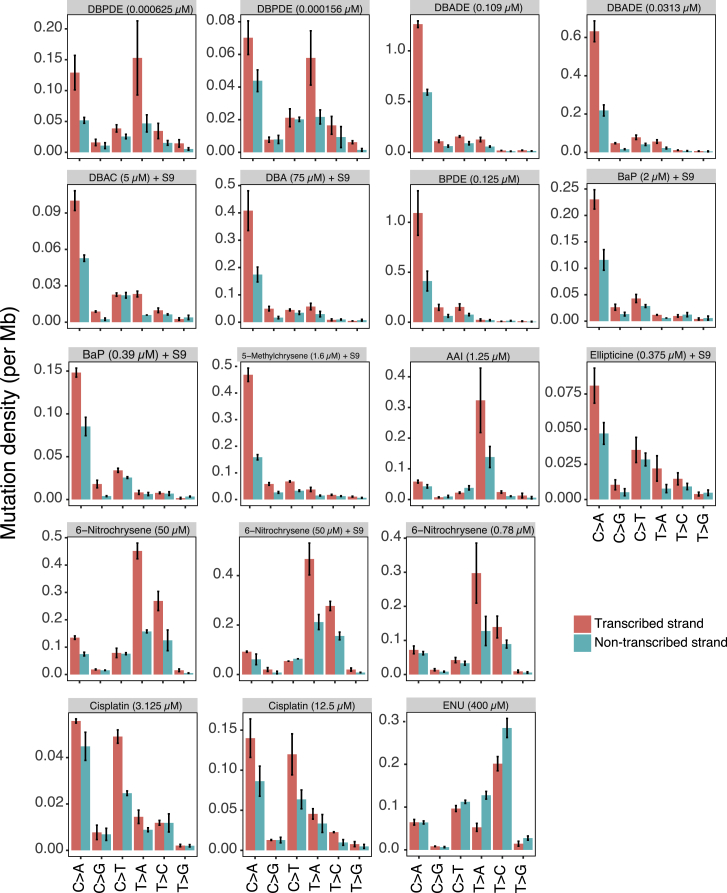
Histogram of Mutation Density on Transcribed (Red) and Non-transcribed (Cyan) Strands of Treatments Having q Value ≤ 0.01, Related to Figure 7 Bars and error represent mean ± SEM of subclone observations.

We found marked transcriptional strand asymmetry for some mutagens, although effect sizes were variable (Figures 7A and S7). Tobacco-related compounds displayed exceptional strand bias particularly in their predominant mutation class (G > A/C > T or T > A/A > T). 6-Nitrochrysene, selected drug therapies, ENU, and AAI also demonstrated transcriptional strand bias, implicating the activity of TC-NER in the repair of damage by these mutagens. Of note, SSR did not show transcriptional strand asymmetry, as observed previously in MEFs (Nik-Zainal et al., 2015). This is likely due to the extremely short experimental UV exposure (∼8 s), in contrast to what transpires *in vivo*.

We assessed transcriptional strand asymmetry and found that it was more marked in early RTD than in late RTD particularly for compounds such as tobacco-related mutagens, e.g., BPDE. This was also observed for cancer-derived signatures associated with tobacco smoking. It suggests that there is greater TC-NER activity in early RTD than in late RTD in human cells in general. In fact, the gradient from early to late is steeper in the *in vitro* system suggesting that TC-NER is fully functional and operating to the same level, if not to a higher level, in iPSCs as *in vivo* (Figures 7B and 7C).

Finally, the background signature of cells grown in culture had a distribution that is more typical of cancer-derived signatures, with more mutations in late RTD (Figure S6). Unlike the short duration of exposure to environmental agents *in vitro*, the cells have had a more uniform and continuous exposure to ROS in culture. Additionally, apart from the components of base excision repair that are required to fix free radical damage, a critical player in maintaining this gradient from early to late RTD is MMR (Supek and Lehner, 2015). MMR-deficient tumors have more mutations in early RTD. Thus, if MMR was not fully competent in iPSCs, we would not see more mutations in late RTD in the background signature.

In summary, DDR, DSB repair, MMR, and TC-NER are fully operative in this cell system. A primary adduct can result in many different mutation outcomes, dependent on which repair pathways respond to the pre-mutagenic state.

## Discussion

We have presented a unique, comprehensive experimental and analytical dataset, documenting the effects of known or suspected environmental mutagens in a single stem cell line. There is a mutational process present in all subclones indicative of stresses irrespective of treatment. Given the identical availability of DNA repair pathways, any additional mutational pattern seen can be attributed to DNA damage associated with each agent.

Notably, the effect size associated with each treatment is variable. Here, we have measured how damaging each environmental mutagen is at comparable levels of cytotoxicity (Figure 2D shows the relative mutagenic capability for each treatment in generating substitution, double-substitution, or indel signatures). Many signatures for which epidemiological relationships are described (UV with melanoma, smoking with lung cancer, and aristolochic acid with urothelial cancers) are attributed to agents that have large effect sizes. For agents with smaller effect sizes, it has been or will be challenging to discern a signal in primary human tumors given the noise from an uncontrolled setting (including multiple endogenous signatures and variable exposure in different people). Thus, it may be more difficult to detect epidemiological relationships with environmental exposures where the mutagenic signal is weak.

Nonetheless, this systematic exercise provides us with a first, extensive resource of *a priori* signatures. The added knowledge may uncover evidence of environmental exposures in human cancers, aiding epidemiological investigations into new causes of cancer. However, this must be done with caution, because depending on the mathematical methods used, supervised fitting of these or other mutational signatures could lead to falsely suggesting exposures where there have not been any. This is important to stress as it may have important legal implications for industries where some of these agents may be occupational exposures.

Could there be differences if these experiments were repeated in different cell types? Perhaps. There may be differences in mutational signatures incurred by normal, primary cells of specific tissues, and differences if similar experiments were conducted in cancer cell lines. DNA damage and, almost certainly, DNA repair could be tissue-dependent. One would also anticipate differences in mutational outcomes when performed on diverse DNA repair defective cellular backgrounds. Notwithstanding, an important point for this study was to use a single cell line with a relatively unmutilated genome as a primary canvas from which to delineate the signature outcomes of environmental agents. Having a cell line where tissue-specific effects and/or selection would not strongly influence the mutagenic outcome was important. Thus, we sought to use an undifferentiated normal cell line to generate a reference set of mutation patterns of environmental mutagens. Human iPSCs have potential for directed differentiation into different tissues in the future and for comparability with other undifferentiated cell lines and/or differentiated tissue-specific 3D cultures. However, for agents that cause hefty mutagenesis (e.g., UV, PAHs, AAI), damage is probably so extensive that the signal may turn out to be essentially the same no matter what system is used.

Finally, we have gained insights into the mechanisms of mutagenesis for several environmental agents, including putative fixation of double-substitutions and indels, and extended our understanding of the contributions of DNA repair pathways (direct reversal, TC-NER, MMR) that are likely operational in iPSCs. Indeed, the absence of rearrangement and copy-number signatures suggests that DDR and cell-cycle checkpoint activity are functioning vigorously, with minimal tolerance of DSBs in sensitive stem cells.

We have demonstrated an assortment of mutational outcomes arising from environmental exposures in a normal stem cell type. The systematic experimental process and standardized analytical steps in this study permits comparability within and across agent families, providing a foundation on which to build further experiments. In order to gain further insights into the mechanisms of mutagenesis, future studies could explore environmental mutagenesis on selected DNA repair defective backgrounds.

## STAR★Methods

### Key Resources Table

**Table:** 

REAGENT or RESOURCE	SOURCE	IDENTIFIER
**Antibodies**		

Rabbit polyclonal anti-phospho CHK2 (Thr68)	Cell Signaling Technology	Cat#2661; RRID:AB_331479
Mouse monoclonal anti-phospho p53 (S15)	Cell Signaling Technology	Cat#9286; RRID:AB_331741
Mouse monoclonal anti-p21	BD Biosciences	Cat#556431; RRID:AB_396415
Rabbit monoclonal anti-phospho Histone H2A.X (S139)	Cell Signaling Technology	Cat#9718; RRID:AB_2118009
Mouse monoclonal anti-Glyceraldehyde-3-PDH (GAPDH)	Millipore	Cat#MAB374; RRID:AB_2107445

**Chemicals, Peptides, and Recombinant Proteins**		

rat liver S9	MolTox	11-101
hamster liver S9	MolTox	15-03SL.5
(±)-anti-Benzo[*a*]pyrene-7,8-dihydrodiol-9,10-epoxide; BPDE	Synthesized at the Institute of Cancer Research (London, UK); Kucab et al., 2015. https://doi.org/10.1016/j.mrfmmm.2015.01.013.	CAS: 55097-80-8
(±)-anti-Dibenz[*a,h*]anthracene-3,4-diol-1,2-epoxide; DBADE	Synthesized by the Biochemical Institute for Environmental Carcinogens (Grosshansdorf, Germany); Wohak et al., 2016. https://doi.org/10.1007/s00204-014-1409-1	CAS: 70951-81-4
(±)-anti-Dibenzo[*a,l*]pyrene-11,12-dihydrodiol-13,14-epoxide; DBPDE	Synthesized by the Biochemical Institute for Environmental Carcinogens (Grosshansdorf, Germany); Wohak et al., 2016. https://doi.org/10.1007/s00204-014-1409-1	CAS: 153926-04-6
1,2-Dimethylhydrazine	Sigma-Aldrich	D161802; CAS: 540-73-8
1,4-Benzoquinone	Santa Cruz	sc-202873; CAS: 106-51-4
1,6-Dinitropyrene	Sigma-Aldrich	284327; CAS: 42397-64-8
1,8-Dinitropyrene	Sigma-Aldrich	284319; CAS: 42397-65-9
1-Nitropyrene	Sigma-Aldrich	N22959; CAS: 5522-43-0
2,6-Dimethylaniline	Sigma-Aldrich	D146005; CAS: 87-62-7
2-amino-1-methyl-6-phenylimidazo[4,5-b]pyridine; PhIP	Synthesized at the Biochemical Institute for Environmental Carcinogens (Grosshansdorf, Germany); Krais et al., 2016. https://doi.org/10.1002/ijc.29836	CAS: 105650-23-5
2-amino-3,8-dimethylimidazo[4,5-f]quinoxaline; MeIQX	Toronto Research Chemicals	A606600; CAS: 77500-04-0
2-amino-3-methyl-9H-pyrido[2,3-b]indole; MeAαC	Toronto Research Chemicals	A617500; CAS: 68006-83-7
2-amino-3-methylimidazo[4,5-f]quinoline; IQ	Toronto Research Chemicals	A616500; CAS: 76180-96-6
2-Naphthylamine	Santa Cruz	sc-209239; CAS: 91-59-8
2-Nitrofluorene	Sigma-Aldrich	N16754; CAS: 607-57-8
2-Nitrotoluene	Sigma-Aldrich	438804; CAS: 88-72-2
3-Nitrobenzanthrone	Synthesized as described by Arlt et al., 2002. PMID: 12419844	CAS: 17117-34-9
4,4’-Methylene(2-chloroaniline)	Tokyo Chemical Industry Co., Ltd.	M0609; CAS: 101-14-4
4-Aminobiphenyl	Sigma-Aldrich	A2898; CAS: 92-67-1
5-Methylchrysene	Sigma-Aldrich	BCR081R; CAS: 3697-24-3
6-Nitrochrysene	Sigma-Aldrich	BCR309; CAS: 7496-02-8
7H-Dibenzo[c,g]carbazole	Sigma-Aldrich	BCR266; CAS: 194-59-2
Acetaldehyde	Sigma-Aldrich	W200301; CAS: 75-07-0
Acrolein	Sigma-Aldrich	89116; CAS: 107-02-8
Acrylamide	Sigma-Aldrich	01700; CAS: 79-06-1
Aflatoxin B1	Sigma-Aldrich	A6636; CAS: 1162-65-8
Aristolochic acid I	Synthesized at the Institute of Cancer Research, London, UK. https://doi.org/10.1007/s00204-016-1808-6.	CAS: 313-67-7
Aristolochic acid II	Synthesized at the Institute of Cancer Research, London, UK. https://doi.org/10.1007/s00204-016-1808-6.	CAS: 475-80-9
AZ20	Tocris Bioscience	5198; CAS: 1233339-22-4
AZD7762	Cayman Chemical	11491; CAS: 860352-01-8
Benzidine	Santa Cruz	sc-214583; CAS: 92-87-5
Benzo[*a*]pyrene	Sigma-Aldrich	B1760; CAS: 50-32-8
Bleomycin	Lundbeck, Ltd.	Lundbeck, Ltd.; CAS: 11056-06-7
Cadmium chloride	Sigma-Aldrich	202908; CAS: 10108-64-2
Camptothecin	Cambridge Bioscience	C0150; CAS: 7689-03-4
Carboplatin	Johnson Matthey, UK	Johnson Matthey, UK; CAS: 41575-94-4
Catechol	Alfa Aesar	10164; CAS: 120-80-9
Cisplatin	Sigma-Aldrich	P4394; CAS: 15663-27-1
Cobalt(II) chloride	Sigma-Aldrich	C8661; CAS: 7646-79-9
Cyclophosphamide	Sigma-Aldrich	C7397; CAS: 50-18-0
Dibenz(a,j)acridine	Sigma-Aldrich	BCR154; CAS: 224-42-0
Dibenz[*a,h*]anthracene	Synthesized by the Biochemical Institute for Environmental Carcinogens; Wohak et al., 2016. https://doi.org/10.1007/s00204-014-1409-1	CAS: 53-70-3
Dibenzo[*a,l*]pyrene	Synthesized by the Biochemical Institute for Environmental Carcinogens; Wohak et al., 2016. https://doi.org/10.1007/s00204-014-1409-1	CAS: 191-30-0
Diethyl sulfate	Alfa Aesar	L14291; CAS: 64-67-5
Dimethyl sulfate	Sigma-Aldrich	D186309; CAS: 77-78-1
Ellipticine (5,11-dimethyl-6H-pyrido[4,3-b]carbazole)	Calbiochem	324688; CAS: 519-23-3
Etoposide	Cayman Chemicals	12092; CAS: 33419-42-0
Formaldehyde	Sigma-Aldrich	F1635; CAS: 50-00-0
Furan	Sigma-Aldrich	185922; CAS: 110-00-9
Glycidamide	Sigma-Aldrich	04704; CAS: 5694-00-8
Hydrogen peroxide	Sigma-Aldrich	H1009; CAS: 7722-84-1
Lead(II) acetate	Alfa Aesar	A11746; CAS: 301-04-2
Lead(II) nitrate	Alfa Aesar	A16345; CAS: 10099-74-8
Mechlorethamine; nitrogen mustard	ApexBio	B1785; CAS: 51-75-2
Melphalan	Sigma-Aldrich	M2011; CAS: 148-82-3
Methyl methanesulfonate	Sigma-Aldrich	129925; CAS: 66-27-3
Methyleugenol	Sigma-Aldrich	284424; CAS: 93-15-2
Mitomycin C	Santa Cruz	sc-3514B; CAS: 50-07-7
Mutagen X (3-Chloro-4-(dichloromethyl)-5-hydroxy-2(5H)-furanone)	Toronto Research Chemicals	C365665; CAS: 124054-17-7
N-Ethyl-N-nitrosourea; N-Nitroso-N-ethylurea	Sigma-Aldrich	N3385; CAS: 759-73-9
Nickel(II) chloride	Alfa Aesar	B22085; CAS: 7791-20-0
N-Methyl-N’-nitro-nitrosoguanidine	Tokyo Chemical Industry Co., Ltd.	M0527; CAS: 70-25-7
N-Methyl-N-nitrosourea; N-Nitroso-N-methylurea	Sigma-Aldrich	N1517; CAS: 684-93-5
N-Nitrosopyrrolidine	Sigma-Aldrich	158240; CAS: 930-55-2
o-Anisidine	Sigma-Aldrich	A88182; CAS: 90-04-0
Ochratoxin A	Sigma-Aldrich	O1877; CAS: 303-47-9
Olaparib; AZD2281	Cayman Chemicals	10621; CAS: 763113-22-0
o-Toluidine	Sigma-Aldrich	185426; CAS: 95-53-4
Peroxynitrite; Peroxynitrous acid, sodium salt	Calbiochem	516620; CAS: 14042-01-4
Potassium bromate	Alfa Aesar	A18258; CAS: 7758-01-2
Potassium chromate	Santa Cruz	sc-203351; CAS: 7789-00-6
Propylene oxide	Sigma-Aldrich	82320; CAS: 75-56-9
Semustine; Methyl-CCNU	Santa Cruz	sc-391062; CAS: 13909-09-6
Sodium (meta)arsenite	Fluka	discontinued; CAS: 7784-46-5
Styrene oxide	Sigma-Aldrich	S5006; CAS: 96-09-3
Sudan I (1-phenylazo-2-hydroxy-naphthalene)	Sigma-Aldrich	51383; CAS: 842-07-9
Temozolomide	Sigma-Aldrich	T2577; CAS: 85622-93-1

**Critical Commercial Assays**		

Deep Blue Cell Viability Kit	Biolegend	Cat# 424702

**Deposited Data**		

Raw and analyzed data	This paper	EGAD00001004583
Mutation data	This paper	http://doi.org/10.17632/m7r4msjb4c.2

**Experimental Models: Cell Lines**		

Human iPSC line	Wellcome Trust Sanger Institute	N/A

**Software and Algorithms**		

Graphpad Prism	Graphpad	SCR_002798
R	R Core Team	https://www.R-project.org/
R codes	This paper	https://github.com/xqzou/Cell_MutagenSig
CaVEMan	Jones et al., 2016	http://cancerit.github.io/CaVEMan/
Pindel	Raine et al., 2015, Ye et al., 2009	http://cancerit.github.io/cgpPindel
BRASS	Nik-Zainal et al., 2016	https://github.com/cancerit/BRASS
IntersectBed	Quinlan and Hall, 2010	https://bedtools.readthedocs.io/en/latest/content/tools/intersect.html

**Other**		

Resource website for the mutagen signatures	This paper	Mutational Signature website, SIGNAL, that is in preparation

### Contact for Reagent and Resource Sharing

Further information and requests for reagents may be directed to, and will be fulfilled by the corresponding authors Serena Nik-Zainal (Lead contact, sn206@cam.ac.uk) and David H. Phillips (david.phillips@kcl.ac.uk).

### Experimental Model and Subject Details

The human iPSC line used for this study was derived at the Wellcome Trust Sanger Institute (Hinxton, UK). The use of this cell line model was approved by Proportionate Review Sub-committee of the National Research Ethics (NRES) Committe North West - Liverpool Central under the project “Exploring the biological processes underlying mutational signatures identified in induced pluripotent stem cell-lines (iPSCs) that have been genetically modified or exposed to mutagens” (ref: 14.NW.0129). It is a long-standing iPSC line originally derived from a patient with alpha-1-antitrypsin deficiency, for which one of the alleles was corrected. The cell line is diploid and does not have any known driver mutations. It does carry a balanced translocation between chromosomes 6 and 8. It is stably growing in culture and does not acquire a vast number of karyotypic abnormalities. This is confirmed through the WGS data reviewed of all 328 subclones. Cell culture reagents were obtained from Stem Cell Technologies, unless otherwise indicated. Cells were routinely cultured and treated on Vitronectin XF-coated plates [10-15 μg/mL] in TeSR-E8 medium at 37°C in 5% CO_2_, with the exception of cells treated in the presence of S9 mix which were seeded on Matrigel-coated plates (Corning). Medium was replenished daily and cultures were passaged at 80% confluency every 3–4 days using Gentle Cell Dissociation Reagent. Frozen stocks were prepared in Knockout Serum Replacement (GIBCO) supplemented with 10% DMSO and stored in liquid nitrogen.

### Methods Details

#### Treatment with DNA damaging agents and DDR inhibitors

The agents examined in this study are listed in Table S1, which includes information on sources, preparation of working stocks and treatment duration. Compounds were dissolved in appropriate solvents where necessary, as shown, and diluted in growth media immediately prior to treating cells. Cells were exposed to treatment media or solvent control media for up to 24 hr, then growth media was replaced and replenished daily as necessary. Compounds requiring cytochrome P450-mediated metabolic conversion to DNA-reactive intermediates were also tested with the inclusion of S9 mix, which consisted of 0.25% S9 fraction from Aroclor-1254-induced male Sprague Dawley rat liver (#11-101) or male Golden Syrian hamster liver (#15-03SL.5) (Moltox), 3 mM NADP (Roche) and 15 mM DL-isocitric acid trisodium salt hydrate (Sigma) in media. Cells were exposed to compounds in the presence of S9 mix for 3 hr, then replaced with fresh growth media. For nonchemical agents, cells were treated as follows: cells were exposed in PBS to simulated solar radiation (SSR) using a 300W-16S xenon arc solar UV simulator (Solar Light, Glenside, USA), as described recently (Lawrence et al., 2018), kindly provided by Professor Antony Young (King’s College London). The SSR output consisted of ≤ 10% UVB (∼295–315 nm) and ≥ 90% UVA (315–400 nm). Cells were gamma-irradiated in media using a Noridon GC-1000S v2.9 cell irradiator, which contains a caesium-137 source delivered at a dose rate of 250 ± 0.59% Gy/hour (access provided by Dr Mahvash Tavassoli, King’s College London).

#### Assessment of cell viability

Cells were seeded for viability assays on 96-well plates, ensuring that cells were dissociated into clumps of ∼10–25 cells in size prior to seeding. For gamma irradiation, cells were seeded into 12.5-cm^2^ flasks. Cells were treated at 5%–10% confluency with a range of concentrations (≥6) for each agent, in order to establish dose-response curves spanning 0%–100% cytotoxicity, where possible, with each treatment condition tested in ≥ 3 technical replicates. Following the appropriate treatment duration, cells were maintained in growth medium to allow cell division and processing of DNA damage. At 72 hr after treatment initiation, cell viability was quantified using the Deep Blue Cell Viability Kit (Biolegend), which measures the reduction of reazurin to fluorescent resorufin by viable cells. Fluorescence (Ex_530_/Em_590_) was measured using a plate reader. Data are presented as the amount of fluorescence of treated cells relative to that of control (media only or solvent-treated) cells and are representative of at least three independent experiments. IC_50_ values were calculated using Prism 7 software.

#### Western blotting

Cells were seeded into 12-well plates and treated with a range of concentrations of each agent, as described above, including a negative control (media only or solvent-treated). At 8 hr or 24 hr post-treatment, cells were washed with PBS and lysed (62.5 mM Tris [pH 6.8], 1 mM EDTA, 2% SDS, 10% glycerol, 1X Halt Protease Inhibitor Cocktail (Thermo Scientific)). β-Mercaptoethanol (0.1% v/v) and bromophenol blue (0.01% w/v) were added to each lysate prior to denaturation at 95°C for 5 min. Cells treated with hydrogen peroxide or gamma irradiation were also harvested 2 or 4 hr post-treatment. Equal amounts of protein (10–20 μg) were loaded onto 4%–12% Bis-Tris gels (NuPAGE; #NP0336 Invitrogen), separated by SDS-PAGE and transferred onto nitrocellulose membranes. Each gel was also loaded with a lysate from cells treated with cisplatin (3.125 μM; 24 h) as a positive control for DDR protein expression. Membranes were incubated with primary antibody (anti-phospho-CHK2 (T68) (#2661 Cell Signaling Technologies), anti-phospho-p53 (S15) (#9286 Cell Signaling Technologies), anti-p21 (#556431 BD PharMingen), anti-phospho-Histone H2A.X (S139) (#9718 Cell Signaling Technologies) and anti-GAPDH (#MAB374 Millipore)) followed by species-specific horseradish peroxidase-conjugated secondary antibody (Bio-Rad) and bands were detected by chemiluminescence.

#### Cell treatment and cloning for WGS

Cells were treated with each agent at a concentration resulting in 40%–60% cytotoxicity, in parallel with cells treated with media only or appropriate solvent control. Additionally, cells were treated with some compounds at a concentration giving > 80% cytotoxicity. Following treatment, cells were cultured for ∼7 days to recover and expand, then frozen stocks were prepared. To isolate single cell clones, treated cell populations were dissociated into single cell suspensions using Accutase (Innovative Cell Technologies) and seeded at limiting dilution on 96-well plates in the presence of 10 μM Y-27632 (Sigma). Medium was replaced daily (without Y-27632) until clones were established (7 – 10 days), then 6 clones were passaged from each treatment condition to 6-well plates for expansion. Frozen stocks were prepared for each clone in addition to a cell pellet for DNA isolation. The IncuCyte was used to ensure that each subclone had arisen from a single cell. This was done by screening images taken every 6 hours over 10-12 days to ensure that: 1) only one single clone is in the well before collection; 2) the single clone does not derive from the ultimate fusion of two clones (at any time point); 3) the single clone derives from a single-cell, defined by the observation of the first division of the cell into two cells. A minimum of three experienced operators reviewed the clones and were required to agree on their observations.

#### DNA extraction and library preparation

Samples were quantified with Biotium Accuclear Ultra high sensitivity dsDNA Quantitative kit using Mosquito LV liquid platform, Bravo WS and BMG FLUOstar Omega plate reader and cherrypicked to 200ng / 120μl using Tecan liquid handling platform. Cherrypicked plates were sheared to 450bp using a Covaris LE220 instrument. Post sheared samples were purified using Agencourt AMPure XP SPRI beads on Agilent Bravo WS. Libraries were constructed (ER, A-tailing and ligation) using ‘NEB Ultra II custom kit’ on an Agilent Bravo WS automation system. KapaHiFi Hot start mix and IDT 96 iPCR tag barcodes were used for PCR set-up on Agilent Bravo WS automation system. PCR cycles include 6 standard cycles: 1) Incubate 95C 5 mins; 2) Incubate 98C 30 s; 3) Incubate 65C 30 s; 4) Incubate 72C 1 min; 5) Cycle from 2, 5 more times; 6) Incubate 72C 10 mins. Post PCR plate was purified using Agencourt AMPure XP SPRI beads on Beckman BioMek NX96 liquid handling platform. Libraries were quantified with Biotium Accuclear Ultra high sensitivity dsDNA Quantitative kit using Mosquito LV liquid handling platform, Bravo WS and BMG FLUOstar Omega plate reader, then pooled in equimolar amounts on a Beckman BioMek NX-8 liquid handling platform and finally normalized to 2.8 nM ready for cluster generation on a c-BOT and loading on requested Illumina sequencing platform. Pooled samples were loaded on the X10 using 150 PE run length, sequenced to 30X coverage. The details of sequence coverage for all clones and subclones are provided in Table S5.

#### Alignment and somatic variant-calling

Human reference genome GRCh37/hg19 was used for short reads alignment. CaVEMan (http://cancerit.github.io/CaVEMan/), Pindel (http://cancerit.github.io/cgpPindel) and BRASS (https://github.com/cancerit/BRASS) were used to call somatic substitutions, indels and rearrangements in all subclones, respectively. Variant allele fraction distribution for each daughter subclone was examined and a filter of VAF ≥ 0.2 was applied to substitutions and indels. Shared mutations among subclones were removed to obtain *de novo* somatic mutations after mutagen treatments. Table S2 summarizes the numbers of *de novo* mutations (substitutions, indels and rearrangements) for all subclones.

### Quantification and Statistical Analysis

#### Identification of background signatures

The mutational profiles of control subclones represent the pattern of background mutagenesis (background signatures). Each control subclone has ∼250 substitutions, ∼10 indels and ∼1 double-substitution. We could neglect the background double-substitution signature from mutagen-treated cells, as the number is close to zero. However, the background substitution and indel mutagenesis are not negligible, we need to identify these signatures in controls in prior to characterize mutagen-associated mutational signatures.

We used 96 and 29 channels to describe substitution and indel profiles, respectively. Hence, the substitution-to-channel ratio is about 2.6, and the indel-to-channel ratio is about 0.34, less than 1. Indeed, this mutation-to-channel ratio affects the profile similarity between control subclones. All the control subclones have very similar substitution profiles (cossim > = 0.9), while their indel profiles show much less consistency (0.08 ≤ cossim ≤ 0.97). Hence, we use the mean of substitution profiles of 35 control subclones as background substitution signature (Figure 3A). For background indel signature, we aggregated 35 control subclone indel profiles, as shown in Figure 5A. To validate the control indel signature, we aggregated indels from treatments which show the same indel burden as controls (and therefore unlikely to have signatures) (p value > 0.1) and indels from treatments which are likely to have signatures (p value < 0.01), as shown in Figure S4. Indeed, aggregated control indel profile (Figure S4C) is almost identical (cosine similarity = 0.99) to the aggregated indel profiles of no-indel-increase treatments (Figure S4D). In contrast, the cosine similarity between control indel profiles and the aggregated indel profiles of treatments with p value < 0.01 (Figure S4B) is 0.78. This result reinforces how the aggregated control indel profile present the full picture of the background indel mutagenesis.

#### Characterization of mutagen-associated mutational signatures

Compared with control cells (no mutagen was applied to cells), mutagen-treated cells not only have background mutagenesis present in the cell, but may also have additional mutagen-associated mutagenesis which increases the mutation burden in the cell (Figure 1C):

(1) $$N_{\mathrm{subclone}}=N_{\mathrm{background}}+N_{\mathrm{mutagen}}.$$

$N_{subclone}$ is the number of mutations observed in mutagen-treated subclones. $N_{\mathrm{background}}$ and $N_{\mathrm{mutagen}}$ are the number of mutations resulting from background and mutagen mutagenesis, respectively. The extra mutation burden $\left(N_{\mathrm{mutagen}}\right)$ is mutagen-dependent–determined by the specific damage of mutagen on DNA and the downstream repair pathways. Different mutational process can result in different combinations of mutational types, termed mutational signatures, which can be expressed as a *K*-vector. For example, background mutagenesis produces a mutational signature $P_{\mathrm{background}}={\left[p_{\mathrm{background}}^{1},p_{\mathrm{background}}^{2},\cdots ,p_{\mathrm{background}}^{K}\right]}^{T}$, where $\sum_{\text{k}=1}^{\text{K}}p_{\mathrm{background}}^{k}=1$, and *K* is the number of mutation types (96 for substitutions and 29 for indels). Similarly, the mutational spectrum (combinations of mutational types) of the extra mutation burden induced by mutagen treatment is defined as mutagen-associated mutational signatures, $P_{\mathrm{mutagen}}={\left[p_{\mathrm{mutagen}}^{1},p_{\mathrm{mutagen}}^{2},\cdots ,p_{\mathrm{mutagen}}^{K}\right]}^{T}$, where $\sum_{\text{k}=1}^{\text{K}}p_{\mathrm{mutagen}}^{k}=1$. There are four steps to characterize mutagen-associated mutational signatures, $P_{mutagen}$.

First, determining the increase of mutation numbers in subclones of mutagen-treated cells. In the present study, every treatment has 2-4 subclones, so the average mutation burden of each treatment can be obtained. Based on 35 control subclones, we constructed the distributions of means of 2, 3 and 4 control subclones using bootstrap resampling techniques. According to the control bootstrapping distribution, the p value of the mutation number for each mutagen treatment can be calculated, and followed by multiple testing correction. Adjusted p value < 0.01 indicating a significant increase of mutation number was observed in a specific mutagen treatment.

Second, measuring the distinction between mutation profile of control and mutagen subclone profiles. The mutation profile of control subclone is $M_{\mathrm{control}}={\left[m_{\mathrm{control}}^{1},m_{\mathrm{control}}^{2},\cdots ,m_{\mathrm{control}}^{K}\right]}^{T}$, where $\sum_{\text{k}=1}^{\text{K}}m_{\mathrm{control}}^{k}=N_{\mathrm{control}}$, and $m_{\mathrm{control}}^{k}$ is the mutation number observed in mutation type k. The mutation profile of mutagen subclone is $M_{\mathrm{subclone}}={\left[m_{\mathrm{subclone}}^{1},m_{\mathrm{subclone}}^{2},\cdots ,m_{\mathrm{subclone}}^{K}\right]}^{T}$, where $\sum_{\text{k}=1}^{\text{K}}m_{\mathrm{subclone}}^{k}=N_{\mathrm{subclone}}$, and $m_{\mathrm{subclone}}^{k}$ is the mutation number observed in mutation type k. We calculated the “signal-to-noise” ratio (*SNR*) between control profiles and mutagen subclone profiles to identify if they have significant difference. ${\bar{M}}_{\mathrm{subclone}}$ and ${\bar{M}}_{control}$ denote the means of the mutation profiles of mutagen subclones and control subclones, respectively; $\sigma_{\mathrm{subclone}}$ and $\sigma_{\mathrm{control}}$ denote the standard deviations of the mutation profiles of mutagen subclones and control subclones, respectively. SNR is calculated through

(2) $$SNR=\frac{{\left\|{\bar{M}}_{\mathrm{subclone}}-{\bar{M}}_{\mathrm{control}}\right\|}_{2}}{\sigma_{\mathrm{mutagen}}+\sigma_{\mathrm{control}}}.$$

The value of *SNR* depends on two components. One is related to signal $\left({\left\|{\bar{M}}_{\mathrm{subclone}}-{\bar{M}}_{\mathrm{control}}\right\|}_{2}\right)$, which measures the averaged Euclidean distance between the mutation profiles of mutagen subclones and control subclones. The other one is related to noise $\left(\sigma_{\mathrm{mutagen}}+\sigma_{\mathrm{control}}\right)$, measuring the variability (consistency) of mutation profiles of subclones. A large value of *SNR* indicates that the difference between the control subclones and mutagen-treated subclones is more likely to be distinguishable from their noises and, therefore, the mutagen-associated signature may be separated from the background signature. The threshold of *SNR* we chose to determine if a mutagen-treatment generates a signature is 2, corresponding to about 90% of mutagen profiles are different from the centroid of control subclone profiles with p value < 0.1.

Third, extracting mutagen-associated mutational signatures by removing the background mutation profile from subclone mutational profile for treatment with p value < 0.01 and *SNR* > = 2. The mutational profile of mutagen-treated subclones is a linear combination of the mutational profile of background mutagenesis and the mutational profile of mutagen-associated mutagenesis:

(3) $$M_{\mathrm{subclone}}=M_{\mathrm{background}}+M_{\mathrm{mutagen}}=N_{\mathrm{background}}\times P_{\mathrm{background}}+N_{\mathrm{mutagen}}\times P_{\mathrm{mutagen}}.$$

Without treating with mutagens, cells only accumulate mutations due to background mutagenesis, so the control mutation profile represents the background mutation profile in control and all mutagen-treated subclones: $M_{\mathrm{background}}≅M_{\mathrm{control}}$, and $N_{\mathrm{background}}≅N_{\mathrm{control}}$. Hence, one can readily derive, using Equation 3,

(4) $$P_{\mathrm{mutagen}}=\frac{\left(M_{\text{subclone}}-M_{\text{control}}\right)}{\left(N_{\mathrm{subclone}}-N_{\mathrm{control}}\right)}.$$

Fourth, measuring the stability of mutational signatures. The stability of a signature is defined as the maximum value of cosine similarity between mutational signatures extracted from each subclone for a given treatment. Stability > = 0.8 indicates a signature was consistent in at least two subclones.

#### Double-substitution mutagenesis

Double substitutions are two substitutions adjacent to each other. With the increase of number of substitutions in a sample, the likelihood of occurrence of two substitutions next to each other increase accordingly. The probability of observing at least one double substitution in a sample is

(5) $$P\left(N_{\text{double}-\text{substitution}}\geq 1\right)=1-P\left(N_{\text{double}-\text{substitution}}=0\right)\;.$$

$N_{\text{double}-\text{substitution}}$ is the number of double substitutions observed in a sample. For each sample, the number of mutations and its mutational profile are known. The 32-trinucleotide frequency in the reference genome can be obtained. For a sample with $N_{\mathrm{sub}}$ substitutions, the probability of occurrence of the first mutation is $P_{1}=1$. The second mutation can occur anywhere other than the position of the first mutation and its neighbor positions. According to the trinucleotide of the first mutation and its neighbors, the available positions $\left(N_{\mathrm{available}\;\mathrm{positions}}\right)$ for the second mutation can be updated and, therefore, the probability of the second mutation can be calculated by $P_{2}=N_{\mathrm{available}\;\mathrm{positions}}/N_{\mathrm{total}\;\mathrm{positions}}$, where $N_{\mathrm{total}\;\mathrm{positions}}$ is the total number of trinucleotides of the second mutation in the genome. This calculation can continue for the last mutation in $N_{\mathrm{sub}}$. Hence one can obtain

(6) $$P\left(N_{\text{double}-\text{substitution}}=0\right)=P_{1}\times P_{2}\times \cdots \times P_{N_{\mathrm{sub}}}.$$

There are 78 unique double-substitution types shown in Table S6.

Many fewer double substitutions were generated in subclones, compared with substitutions and indels. Hence, in order to appreciate a double-substitutions signature in a pattern of 78 channels, we only consider the treatments which have more than 20 double-substitutions.

#### Genomic features of mutagen-associated signatures

The influence of several genomic features on the experimentally-generated mutational signatures was investigated, including replicative and transcriptional strand bias, the distribution of mutations on replication-timing regions and methylation status on PAH mutagenesis. Reference information of replicative strands and replication-timing regions were obtained from the ENCODE project Repli-seq data (https://www.encodeproject.org/). The transcriptional strand coordinates were obtained from the footprints of protein coding genes in the genome. CpG island coordinates were obtained from Gardiner-Garden’s work (Gardiner-Garden and Frommer, 1987). IntersectBed was used to identify mutations overlapping certain genomic features. Pearson’s chi-square test was used to evaluate the significance of strand bias. All statistical analysis were performed in R. All plots were generated by ggplot2.

### Data and Software Availability

The accession number for the raw sequencing data reported in this paper is EGA:EGAD00001004583. All mutation data can be obtained on Mendeley: http://doi.org/10.17632/m7r4msjb4c.2.

R codes are available at https://github.com/xqzou/Cell_MutagenSig.

### Additional Resources

The curated data will become available for general browsing, down to individual subclone level from our reference Mutational Signature website, SIGNAL, that is in preparation.

## Supplemental Information

Table S1. Details of Treatment Conditions and Outcomes of Functional Assays (Phospho-CHK2, Phospho-p53, and γ-H2AX) of All Environmental Mutagens Used in These Experiments, Related to Figures 1 and S1 and STAR Methods

Table S2. Summary of Number of *De Novo* Somatic Mutations, Including Substitutions, Indels, and Rearrangements in All Subclones, Related to Figure 2

Table S3. Summary of Number of Double Substitutions in All Subclones, Related to Figure 2

Table S4. List of Agents that Gave Rise to a Mutational Signature in Human iPSCs, with Information on Their Pathways of Metabolic Activation, Interactions with DNA, Characteristics of Point Mutations and Similarities, and Differences of Mutational Signatures, Related to Figure 3

Table S5. Sequence Coverage Statistics for All Samples, Related to STAR Methods

Table S6. Seventy-Eight Unique Double-Substitution Types, Related to STAR Methods

Video Abstract
