# Supplementary material for: A Compendium of Mutational Signatures of Environmental Agents
Source: Cell. 2019 May 2;177(4):821–836.e16. doi: 10.1016/j.cell.2019.03.001 (PMC6506336; doi:10.1016/j.cell.2019.03.001)
Supplement: Table S4. List of Agents that Gave Rise to a Mutational Signature in Human iPSCs, with Information on Their Pathways of Metabolic Activation, Interactions with DNA, Characteristics of Point Mutations and Similarities, and Differences of Mutational Signatures, Related to Figure 3 [file mmc4.docx]

**Supplementary Table 4**. List of agents that gave rise to a mutational signature in human iPSCs, with information on their pathways of metabolic activation, interactions with DNA, characteristics of point mutations where known and similarities and differences of mutational signatures.

| Agent | Active metabolite(s) | Known interactions with DNA | Characteristics of mutational signature | Resemblances (and notable differences) to other signatures. Cosine similarity values shown in parentheses |
| --- | --- | --- | --- | --- |
| Aldehydes |  |  |  |  |
| Formaldehyde   | [Direct acting] | Most abundant adduct *in vitro* is N^6^-hydroxymethyl-dA. Also forms interstrand crosslinks in DNA | 57% of mutations at GC; 43% at AT. 28% of mutations are GC-TA; 21% are AT-GC | Not significantly similar to any other agents |
| Alkylating agents |  |  |  |  |
| N-Methyl-N-nitrosourea (MNU)   | [Direct acting methylating agent] | Forms mostly N7 guanine adducts (65-70%; non-mutagenic), but also N3-dA (8-9%; non-mutagenic) O^6^-dG (6-9%) and low amounts of O^2^-dT and O^4^-dT (<1% each) | 89% of mutations are at AT. O^6^-alkylguanine adducts cause GC-AT (18%); O^4^-alkylthymine adducts cause AT-GC (78%); O^2^-alkylthymine adducts cause AT-TA (5%); GC-AT accounts for a further 8% | temozolomide (0.99)  ENU (0.86)  COSMIC signature 12 (0.84) COSMIC signature 26 (0.87) |
| N-Ethyl-N-nitrosourea (ENU)   | [Direct acting ethylating agent] | Forms N7 guanine adducts (~11%; non-mutagenic), N3-dA (2-6%; non-mutagenic) O^6^-dG (7-10%) and O^2^-dT (~7%) and O^4^-dT (1-3%) | 75% of mutations are a AT. O^6^-alkylguanine adducts cause GC-AT (20%); O^4^-alkylthymine adducts cause AT-GC (50%); O^2^-alkylthymine adducts cause AT-TA (20%) | temozolomide (0.87)  MNU (0.86)  COSMIC signature 12 (0.80) COSMIC signature 26 (0.81) |
| Dimethyl sulfate (DMS)   | [Direct acting methylating agent] | Forms N7-dG (non-mutagenic), N3-dA (non-mutagenic), N1-dA and N3-dC and low amount of O^6^-dG | O^6^-alkylguanine adducts cause GC-AT (29%); AT-TA accounts for 26% | DES (0.80)  propylene oxide (0.80) |
| Diethyl sulfate (DES)   | [Direct acting ethylating agent] | Forms N7-dG (67%; non-mutagenic), N3-dA (10%; non-mutagenic) and low amount of O^6^-dG (0.2%) | O^6^-alkylguanine adducts cause GC-AT (40%); AT-TA accounts for 24% | propylene oxide (0.90)  DMS (0.86)  N-nitrosopyrrolidine (0.86)  mechlorethamine (0.82) |
| 1,2-Dimethylhydrazine (DMH)   | [Methylating agent] | Forms adducts similarly to other alkylating agents, with O-alkylated bases presumed to be premutagenic | AT-GC accounts for 33%; GC-AT for 42%; AT-TA for 11% | Not significantly similar to other alkylating agents |
| Drug therapy |  |  |  |  |
| Temozolomide (see also alkylating agents)   | [Direct acting agent] | Forms adducts at N7-dG (non-mutagenic) and O^6^-dG; presumably also alkylates O^2^-dT and O^4^-dT | O^6^-alkylguanine adducts cause GC-TA, but signature is dominated by AT-GC (85%) | MNU (0.99)  ENU (0.87).  Signature is *unlike* that of COSMIC signature 11 (0.16), but bears similarity to signature 12 (0.83), signature 21 (0.84) and signature 26 (0.89) |
| Cyclophosphamide   | Acrolein and phosphoramide mustard | Acrolein forms 8-hydroxy-1,N^2^-propano-dG and 6-hydroxy-1,N^2^-propano-dG; phophoramide mustard forms an unstable N7-dG adduct, which converts to a product that crosslinks with another guanine in DNA | 47% of mutations are GC-TA; 24% are GC-AT | ochratoxin A (0.87) |
| Mechlorethamine (nitrogen mustard)   | [Direct acting agent] | Forms monoadducts at N7-dG (53%) and N3-dA (26%), and a guanine-guanine crosslink (19%) in GNC sequences | Signature is dominated by GC-AT mutations (57%) | propylene oxide (0.89)  N-nitrosopyrrolidine (0.88)  DES (0.82)  COSMIC signature 30 (0.80) |
| Semustine (Methyl-CCNU)   | [Direct acting agent] | See nitrosoureas (above). Adducts formed at N7-dG and O^6^-dG | 58% of mutations are GC-AT | Not significantly similar to any other agents |
| Cisplatin   | [Direct acting agent] | Reacts with N7 positions in guanine and adenine and forms intrastrand (80%) and interstrand (6%) crosslinks, plus dG monoadduct (6%). Adducts formed will be identical to those of carboplatin | 75% of mutations are at GC, and 43% are GC-AT | carboplatin (0.95) |
| Carboplatin   | [Direct acting agent] | Reacts with N7 positions in guanine and adenine and forms intrastrand and interstrand crosslinks. Adducts formed will be identical to those of cisplatin | 76% are at GC, and 44% are GC-AT | cisplatin (0.95) |
| Ellipticine   | 12-hydroxyellipticine and 13-hydroxyellipticine | React with dG in DNA, presumed at N^2^ position | 70% are at GC; 43% of mutations are GC-TA, 19% are GC-AT | benzidine (0.88)  MX (0.87)  3-NBA (0.86)  1,8-DNP (0.84)  BaP (0.82)  COSMIC signature 4 (0.82) COSMIC signature 8 (0.84) |
| DNA damage response inhibitors |  |  |  |  |
| AZD7762   | Not known | A kinase inhibitor; unlikely to react directly with DNA | 87% of mutations are at AT; 59% are AT-TA, 22% are AT-GC | DBP/DBPDE (0.89)  6-nitrochrysene (0.89)  aristolochic acid I (0.88)  COSMIC signature 22 (0.87)  COSMIC signature 25 (0.83) |
| Aromatic amines |  |  |  |  |
| Benzidine   | N-Hydroxylamine, which is converted to reactive N-O-ester | Forms adducts at C8-dG and N^2^-dG | 90% of mutations are at GC; 61% are GC-TA | MX (0.96)  ellipticine (0.88)  BaP (0.85)  PhIP (0.84)  3-NBA (0.83)  1,8-DNP (0.82)  furan (0.82)  DBA (0.81)  COSMIC signature 4 (0.86) |
| 4-Aminobiphenyl (4-ABP)   | N-Hydroxylamine, which is converted to reactive N-O-ester | Forms adducts at C8-dG and N^2^-dG | 64% of mutations are at GC; 36% are GC-TA, 23% are GC-AT, 20% are AT-TA | Not significantly similar to any other agents |
| Heterocyclic amines |  |  |  |  |
| 2-amino-1-methyl-6-phenylimidazo[4,5-*b*]pyridine (PhIP)   | N-Hydroxylamine, which is converted to reactive N-O-ester | Mainly C8-dG adduct formed | 96-98% of mutations are at GC, with signature dominated by GC-TA mutations (76-83%) | BaP/BPDE (0.95)  5-methylchrysene (0.91)  1,8-DNP (0.90)  MX (0.87)  DBAC (0.85)  benzidine (0.84)  COSMIC signature 4 (0.85) |
| Radiation |  |  |  |  |
| Simulated solar radiation (SSR) | 95% UVA  5% UVB | Forms mainly intrastrand pyrimidine dimers: cyclobutadipyrimidines and pyrimidine(6-4)pyrimidones | 87% of mutations are GC-AT | Very good match with signature in UV-treated MEFs and with COSMIC signature 7 (0.94). also similar to COSMIC signature 11 (0.83) |
| Nitrosamines |  |  |  |  |
| N-Nitrosopyrrolidine   | α-hydroxylation leads to multiple reactive metabolites, including crotonaldehyde, 3-hydroxybutanal, paraldol and acetaldehyde | A total of 69 structurally distinct and diverse DNA adducts have been identified, including a number of cyclic adducts | Most mutations are GC-AT (69%), followed by AT-TA (14%) | mechlorethamine (0.88)  propylene oxide (0.88)  DES (0.86)  COSMIC signature 30 (0.80) |
| PAHs/nitro-PAHs |  |  |  |  |
| Benzo[*a*]pyrene (BaP)    Benzo[*a*]pyrene diol-epoxide (BPDE)^1^   | BPDE is the reactive intermediate of BaP | Forms adducts principally with N^2^-dG. Very little reaction with dA. | Major point mutation predicted to be GC-TA. For BaP 91-92% are at GC, and 71-73% are GC-TA. For BPDE 96% are at GC, and 75% are GC-TA, as expected | BaP and BPDE signatures very similar (0.99)  1,8-DNP (0.93)  5-methylchrysene (0.92)  DBA/DBADE (0.91)  PhIP (0.95)  MX (0.90)  benzidine (0.85)  3-NBA (0.85)  ellipticine (0.82)  aflatoxin B1 (0.81)  COSMIC signature 4 (0.89) |
| Dibenz[*a,h*]anthracene (DBA)    Dibenz[*a,h*]anthracene diol-epoxide (DBADE)^2^  ^^ | DBADE is the major reactive intermediate of DBA, but a bis-diol-epoxide is also formed and can contribute to DNA adduct formation | Sites of modification of DNA not known but predicted to involve N^2^-dG and probably N^6^-dA. | Major point mutation predicted to be GC-TA. For DBA 87% are at GC, and 70% are GC-TA. For DBADE 90-91% are at GC, and 74-77% are GC-TA, as expected | DBA and DBADE signatures very similar (0.99)  5-methylchrysene (0.97)  1,8-DNP (0.93)  BaP/BPDE (0.91)  DBAC (0.85)  PhIP (0.85)  benzidine (0.81)  3-NBA (0.87)  MX (0.88)  COSMIC signature 4 (0.87) |
| Dibenzo[*a,l*]pyrene (DBP)^3^    Dibenzo[*a,l*]pyrene diol-epoxide (DBPDE)^4^   | DBPDE is the major reactive intermediate of DBP | Forms adducts more predominantly with N^6^-dA than with N^2^-dG | DBP signature is 15-30% at GC, with 33-70% AT-TA. DBPDE signature is 26-33% at GC, 67-74% at AT; 53-60% of mutations are AT-TA, 18-23% are GC-TA | DBP and DBPDE signatures very similar (0.96)  Also to be noted is the similarity of the AT-TA section of the profile to that of AAI (0.96)  6-nitrochrysene (0.82)  AZD7762 (0.89)  COSMIC signature 22 (0.96)  COSMIC signature 25 (0.82) |
| 5-methylchrysene   | Most likely activated via a diol-epoxide | 5-MeC diol-epoxides react with dG to an extent only slightly less than for BPDE | 90% of mutations are at GC. 71% are GC-TA | BaP/BPDE (0.92)  DBA/DBADE (0.97)  1,8-DNP (0.93)  PhIP (0.91)  3-NBA (0.85)  COSMIC signature 4 (0.86) |
| Dibenz[*a,j*]acridine (DBAC)   | A PAH with a nitrogen atom in one of its 6-membered rings. Could/should be activated via a diol-epoxide | DNA adducts not identified, but at least 2 formed *in vivo* | 70% of mutations are at GC; 52% are GC-TA, 21% are AT-TA | 3-NBA (0.83)  1,8-DNP (0.80)  DBA/DBADE (0.85) |
| 6-nitrochrysene   | Both nitroreduction to N-hydroxy-6-aminochrysene and ring oxidation to 6-nitrochrysene-1,2-dihydrodiol (leading to diol-epoxide) | Major reaction of hydroxylamine with C8 of dA, resulting in N-(deoxyinosin-8-yl)-6-aminochrysene, after deamination of adenine. Also reaction with N^2^-dG and minor reaction with C8 of dG. Diol-epoxide reaction with dG, presumably at N^2^ | In the absence of S9, mutations are overwhelmingly at AT (88%). In the presence of S9, there is a shift towards GC (30%). Predominant mutation in the absence of S9 is AT-TA (54%) but is AT-GC (41%) in the presence of S9. Signatures cluster closely, however. | DBP/DBPDE (0.82)  AZD7762 (0.89)  aristolochic acid II (0.87)  COSMIC signature 16 (0.81)  COSMIC signature 25 (0.81) |
| 1,6-dinitropyrene (1,6-DNP)    1,8-dinitropyrene (1,8-DNP)   | Nitroreduction of one nitro group. Leads to acid-catalysed DNA binding or conversion to highly reactive O-acetyl metabolites | In both cases C8-substituted dG adducts are major products. Minor reaction with dA also proposed | For both compounds most mutation is at GC (84-86% for 1,6-DNP, 86% for 1,8-DNP). For 1,8-DNP GC-TA predominates (50-60%); for 1,6-DNP GC-TA is 34%, GC-AT is 29%, GC-CG is 21%. | Signatures are fairly similar to each other (0.84). 1,8-DNP signature similar to that of  3-NBA (0.97)  BaP/BPDE (0.93)  DBA/DBADE (0.93)  5-methylchrysene (0.93)  PhIP (0.90)  MX (0.87)  ellipticine (0.84)  benzidine (0.82)  DBAC (0.80)  COSMIC signature 4 (0.88) |
| 3-nitrobenzanthrone (3-NBA)   | Activation via nitroreduction | Adducts with C8 and N^2^ of dG and with N^6^ of dA | Mutations are 69-70% at GC, with 45-48% GC-TA | 1,8-DNP (0.87)  BaP/BPDE (0.86)  DBA/DBADE (0.85)  5-methylchrysene (0.85)  MX (0.84)  DBAC (0.83)  benzidine (0.83)  ellipticine (0.82)  COSMIC signature 4 (0.83) |
| ROS |  |  |  |  |
| Potassium bromate  KBrO_3_ | Generates ROS, including hydroxyl radical **^.^**OH | Many DNA adducts potentially formed. 8-OH-dG, also called 8-oxo-dG, will be the most prominent | 8-OH-dG gives rise to GC-TA transversions. 92% of mutations are at GC, and 88-89% are GC-TA. | Signature is similar to COSMIC signature 18 (0.85) |
| Diet/tobacco/drinking water, other compounds |  |  |  |  |
| Aristolochic acid I (AAI)   | aristolactam nitrenium ion | Adducts formed mainly with N^6^-dA; lesser formation of N^2^-dG adduct | AT-TA transversions known from *TP53* and in vitro studies. Signature: 89% of mutations are a AT; 83% are AT-TA. | *Dissimilar* to aristolochic acid II (0.51). Very close match with signature in MEFs and with COSMIC signature 22 (0.99). Note similarity of AT-TA portion of DBPDE signature, however (0.96) AZD7762 (0.88) |
| Aristolochic acid II (AAII)   | aristolactam nitrenium ion. Structure differs from that formed by AAI only in having side chain R = OCH_3_ replaced by R = H | Adducts formed mainly with N^6^-dA; lesser formation of N^2^-dG adduct | 67% of mutations are at AT. AT-TA accounts for 31% of mutations, AT-GC for 28%, GC-AT for 21% | Despite similarity of DNA damage with that of AA1, signature is very different from that of AAI (0.51) and COSMIC signature 22 (0.51)  6-nitrochryene (0.87) |
| Aflatoxin B1 (AFB1)   | aflatoxin 8,9-oxide | Adducts formed with N7-dG; these are unstable and can undergo depurination, leaving an apurinic site in DNA, or imidazole ring opening to yield a stable adduct | 96% of mutations are at GC. 59% are GC-TA, 26% are GC-CG | BPDE (0.81)  Similarity to COSMIC signature not strong (0.68) |
| Ochratoxin A (OTA)   | Not known | Evidence for adduct formation is equivocal | 86% of mutations are at GC. 58% are GC-TA, 21% are GC-AT | methyleugenol (0.88)  cyclophosphamide (0.87) |
| Propylene oxide   | Direct acting | Major site is N7-dG, but also modifies N3-dA (11%), N3-dC (10%) and O^6^-dG (<0.4%) | Signature is 44% GC-AT, 32% AT-TA. | mechlorethamine (0.88)  N-nitrosopyrrolidine (0.88)  DES (0.86)  DMS (0.80) |
| Furan   | cis-2-butene-1,4-dial (BDA) | Forms several cyclic adducts with DNA bases: 1,N^2^-dG, 3,N^4^-dC and 1,N^6^-dA | 89% of mutations are at GC. 51% are GC-TA, 20% are GC-AT, 18% are GC-CG | benzidine (0.82)  MX (0.80) |
| Methyleugenol   | 1’-sulphöoxymethyleugenol | By analogy with other alkenylbenzenes, forms adducts at N^2^-dG and N^6^-dA | 82% of mutations are at GC. 58% are GC-TA | ochratoxin A (0.88) |
| 3-Chloro-4-(dichloromethyl)-5-hydroxy-2(5H)-furanone (MX)   | Direct acting | Reacts with primarily with dG, minor reaction with dA; evidence also for depurination. Forms a cyclic 1,N^2^-dG adduct | 94% of mutations were at GC. Signature had 69% GC-TA, 15% GC-AT | benzidine (0.96)  BaP/BPDE (0.90)  PhIP (0.87)  1,8-DNP (0.87)  ellipticine (0.87)  3-NBA (0.84)  furan (0.80)  COSMIC signature 4 (0.89)  COSMIC signature 24 (0.80) |

^1^ BPDE: (±)-*anti*-benzo[*a*]pyrene-7,8-dihydrodiol 9,10-oxide; one enantiomer is shown

^2^ DBADE: (±)-*anti*-dibenz[*a,h*]anthracene-3,4-dihydrodiol 1,2-oxide; one enantiomer is shown

^3^ Also called dibenzo[*def,p*]chrysene

^4^ DBPDE: (±)-*anti*-dibenzo[*a,l*]pyrene-11,12-dihydrodiol 13,14-oxide; also called (±)-*anti*-dibenzo[*def,p*]chrysene-11,12-dihydrodiol 13,14-oxide; one enantiomer is shown
