# Supplementary material for: A Compendium of Mutational Signatures of Environmental Agents
Source: Cell. 2019 May 2;177(4):821–836.e16. doi: 10.1016/j.cell.2019.03.001 (PMC6506336; doi:10.1016/j.cell.2019.03.001)
Supplement: Table S6. Seventy-Eight Unique Double-Substitution Types, Related to STAR Methods [file mmc6.pdf]

Ref>Alt		Alt															
		AA	AC	AG	AT	CA	CC	CG	CT	GA	GC	GG	GT	TA	TC	TG	TT
Ref	AA																
	AC																
	AG																
	AT																
	CA																
	CC																
	CG																
	GA																
	GC																
	TA																

Table S6. Seventy-eight unique double-substitution types, Related to STAR Mthods. The reference and altered sequences are shown in column and row, respectively. Equivalent reference sequences are combined together, e.g., AA is equivalent to TT. Unqualified double-substitutions are shown in grey, e.g., AA>AC is not a double-substitution. Double-substitutions with different reference sequence are shown in different colours, and the colour code is the same as in Figure 4. White indicates that equivalent ones have been included, e.g., AT>GG is equivalent to AT>CC and has been included, so AT>GG is marked in white.
